# NOD1 is a key mediator of atrial myopathy in heart failure

**DOI:** 10.7150/thno.134756

**Published:** 2026-06-04

**Authors:** Marta Gil-Fernández, Almudena Val-Blasco, Andrea Bueno-Sen, Paula Cantolla-Pablo, Carlos Galán-Arriola, Ali Ayaon, Teresa López-Fernández, Yuriana Aguilar-Sanchez, Satadru K Lahiri, José Alberto Navarro-García, Rafael Peinado, Angel Aroca, Miguel Ángel Rubio, José Antonio Blázquez, María Tamayo, Eduardo López-Collazo, Gema Ruiz-Hurtado, Inmaculada Jorge, Jesús Vázquez, Patricia Prieto, Tarik Smani, Antonio Ordoñez, David Filgueiras-Dama, Raúl Moreno, Borja Rivero-Santana, Jorge Nuche, José Jalife, Borja Ibáñez, Carmen Delgado, Lisardo Boscá, Xander H.T. Wehrens, María Fernández-Velasco

**Affiliations:** 1 Hospital La Paz Institute for Health Research (IdiPAZ), Madrid, Spain.; 2 Spanish National Centre for Cardiovascular Research (CNIC), Madrid, Spain.; 3 La Paz University Hospital, Madrid, Spain.; 4 Cardiovascular Research Institute, Baylor College of Medicine, Houston, TX, USA.; 5 Salamanca University Hospital (CAUSA), Salamanca, Spain.; 6 Sols-Morreale Biomedical Research Institute (IIBM, CSIC-UAM), Madrid, Spain.; 7 Cardiorenal Translational Laboratory, Research Institute Hospital 12 de Octubre (i+12), Madrid, Spain.; 8 Pharmacology Department, Pharmacy Faculty, Complutense University of Madrid (UAM), Spain.; 9 Cardiovascular Pathophysiology group, Institute of Biomedicine of Seville (IBiS), Virgen del Rocío University Hospital /University of Seville/CSIC, Seville, Spain.; 10 Department of Physiology, Autonomous University of Madrid, Spain.; 11 Cardiology Department, Fundación Jiménez Diaz University Hospital Health Research Institute (IIS-FJD), Madrid, Spain.; 12 Cardiovascular Biomedical Research Centre Network (CIBERCV), Madrid, Spain.; 13 Department of Integrative Physiology, Baylor College of Medicine, Houston, TX, USA.; 14 Department of Medicine, Baylor College of Medicine, Houston, TX, USA.; 15 Departments of Medicine and Molecular & Integrative Physiology, University of Michigan, Ann Arbor (J.J.), USA.; 16 Cardiology Department, 12 de Octubre University Hospital, Research Institute Hospital 12 de Octubre (i+12), Madrid, Spain.

**Keywords:** NOD1, atrial remodeling, heart failure, innate immunity, CaMKII, RyR2

## Abstract

Heart failure (HF)-associated atrial myopathy is driven by complex and poorly understood mechanisms. Emerging evidence suggests that innate immune components contribute to atrial remodeling, yet the role of nucleotide-binding oligomerization domain-containing protein 1 (NOD1) receptors remains unclear.

**Methods:**

NOD1 expression was characterized in atrial myocardium of HF patients (n = 36) and non-failing controls (n = 45) undergoing valve surgery, and in two porcine models of atrial myopathy with divergent ventricular phenotypes: aortic banding (AoB; preserved LVEF) and left atrial infarction (LAI; reduced LVEF). The causal role of NOD1 in atrial remodeling was assessed using genetic (*Nod1^⁻/⁻^)* and pharmacological (ML-130) loss-of-function approaches in a murine transverse aortic constriction (TAC) model, and through selective NOD1 activation with C12-iE-DAP in wild-type, *Nod1^⁻/⁻^*, and *RyR2*-S2814A mice. Proteomic, phosphoproteomic, transcriptomic, and Ca²⁺ imaging analyses were performed across experimental systems.

**Results:**

NOD1 was markedly upregulated in atrial myocardium of HF patients and in both porcine models, across divergent ventricular phenotypes and irrespective of documented rhythm status, correlating with structural and functional indices of atrial disease severity. Genetic NOD1 deficiency in TAC mice prevented atrial dysfunction, structural remodeling, activation of profibrotic molecular pathways and Ca²⁺ mishandling. Pharmacological NOD1 inhibition with ML-130 reproduced the protective effects on atrial structural and Ca²⁺ handling. Selective NOD1 activation with C12-iE-DAP induced atrial Ca²⁺ dysregulation through CaMKII-dependent RyR2-Ser2814 hyperphosphorylation, effects that were abrogated by CaMKII inhibition, and absent in *RyR2*-S2814A mice. Human atrial transcriptomic analysis confirmed enrichment of inflammatory, Ca²⁺-signaling, and extracellular matrix remodeling pathways in HF. Increased CaMKII phosphorylation in human atrial myocardium further corroborated the translational relevance of the NOD1–CaMKII–RyR2 axis.

**Conclusions:**

These findings identify the NOD1–CaMKII–RyR2 axis as a cardiomyocyte-centered mechanism linking innate immune activation to atrial Ca²⁺ dysregulation and structural remodeling in HF, establishing NOD1 as a molecular indicator of atrial myopathy burden and a mechanism-based therapeutic target.

## Introduction

Heart failure (HF) is a complex syndrome and a leading cause of global morbidity and mortality. Chronic elevation of left ventricular (LV) filling pressures promotes maladaptive remodeling of the left atrium (LA), characterized by chamber enlargement and functional impairment 1. While ventricular remodeling has been extensively studied 2,3, the molecular mechanisms driving atrial myopathy remain incompletely defined.

Atrial myopathy encompasses structural and functional alterations with substantial clinical implications 4. It may occur independently of atrial fibrillation (AF) 4, although atrial remodeling creates an arrhythmogenic substrate that facilitates AF initiation and maintenance 5,6. Approximately half of patients with chronic HF exhibit moderate to severe LA enlargement, a powerful predictor of all-cause mortality, cardiovascular death, and hospitalization 7. Advances in cardiac imaging, particularly speckle-tracking echocardiography, have enabled detailed evaluation of atrial reservoir, conduit, and contractile function 8,9. Strain-based indices derived from these assessments have emerged as robust prognostic markers in HF, identifying subclinical atrial dysfunction that precedes overt remodeling 10. These observations highlight the clinical relevance of identifying the molecular mechanisms underlying atrial dysfunction to refine risk stratification and enable targeted therapy in HF.

Inflammation and innate immune activation are increasingly recognized as key contributors to HF-associated atrial myopathy 11,12. Pattern recognition receptors (PRRs) detect pathogen-associated and damage-associated molecular patterns (PAMPs and DAMPs), activating inflammatory cascades that can compromise cardiac homeostasis 13,14. Among PRRs, nucleotide-binding oligomerization domain (NOD)-like receptors (NLRs) have emerged as critical regulators of myocardial inflammation, arrhythmogenesis, and HF progression 15-19. Persistent innate immune activation in HF drives atrial inflammation and disrupts intracellular Ca²⁺ handling, contributing to atrial dysfunction and remodeling 15. While the NLRP3 inflammasome is a well-established contributor to atrial remodeling and AF 13,14, the role of nucleotide-binding oligomerization domain-containing protein 1 (NOD1) in HF-associated atrial myopathy remains unclear. NOD1 is a cytosolic PRR that, upon activation, recruits receptor-interacting serine/threonine-protein kinase 2 (RIP2), triggering an NF-κB-dependent inflammatory response 18.

This study identifies NOD1 as a mechanistic contributor to atrial myopathy by integrating human atrial tissue analysis with large-animal and murine HF models. Mechanistically, NOD1 activation enhances CaMKII-dependent phosphorylation of RyR2, promoting sarcoplasmic reticulum Ca²⁺ leak and driving progressive atrial damage. These findings point to NOD1 as a new mechanistic pathway involved in atrial remodeling and a potential therapeutic target in HF-associated atrial myopathy, highlighting its role in connecting innate immune signaling with Ca²⁺ dysregulation and structural remodeling.

## Materials and Methods

### Study approval

The human study protocol was approved by the Human Ethics Committee of La Paz University Hospital (CEIm: 1729) and conducted in accordance with the principles of the Declaration of Helsinki. All participants provided written informed consent prior to inclusion. All animal experiments were approved by the General Direction of Agriculture and Environment and conducted in accordance with Spanish regulations and EU Directive 2010/63/EU (PROEX 103/19 for pigs and PROEX 053/18 for mice).

### Human studies

The cohort included 78 patients undergoing valve surgery: 42 non-failing patients (NF; NT-proBNP < 200 pg/mL, LVEF > 50%) and 36 with HF (including HFmrEF and HFrEF; NT-proBNP ≥ 200 pg/mL, LVEF ≤ 50%). Detailed clinical and echocardiographic parameters are presented in Table 1.

Left or right atrial (LA and RA) appendages were collected intraoperatively, snap-frozen, and stored at -80 °C or processed for molecular, transcriptomic, and histological analyses. Echocardiographic studies were performed according to the American Society of Echocardiography (ASE) and European Association of Cardiovascular Imaging (EACVI) guidelines. Further details on clinical, imaging, and RNA sequencing procedures are provided in the Supplementary Methods.

### Animal studies

#### Swine models

Two independent models were used to induce atrial remodeling secondary to mechanical overload or ischemic injury: (1) aortic banding (AoB) was performed in male Yucatan minipigs (*n =* 13), inducing progressive pressure overload with preserved LVEF; and (2) left atrial infarction (LAI) in male Large White pigs (*n =* 21), resulting in atrial dysfunction with reduced LVEF.

A subset of LAI animals (*n =* 6) underwent dual-chamber pacemaker implantation and atrioventricular (AV) node ablation to enable controlled AF induction by burst pacing. Sham-operated animals served as controls for AoB (*n =* 17) and LAI (*n =* 14). Animals were monitored for 32 weeks (AoB model) or 8 weeks (LAI model) post-surgery, after which LA tissue was collected for analysis.

Cardiac magnetic resonance (CMR) imaging was performed to evaluate cardiac function, including reservoir, conduit, and contractile function. Detailed surgical and imaging protocols are described in the Supplementary Methods.

#### Mouse studies

Transverse aortic constriction (TAC) was performed in male wild-type (WT) and *Nod1^⁻/⁻^* mice on a C57BL/6J background to induce pressure overload. Sham-operated mice underwent identical procedures without constriction. Successful *Nod1* knockout was confirmed by genotyping (Figure S1). Atrial tissue was collected 4 weeks after TAC.

A subset of WT-Sham and WT-TAC mice was treated with the selective NOD1 inhibitor ML-130 (ML; 2 mg/kg, i.p.) every other day for 4 weeks. Pharmacological studies further comprised daily intraperitoneal administration of saline or the NOD1 agonist C12-iE-DAP (iE; 3.3 mg/kg, i.p.) in WT, *Nod1^⁻/⁻^,* and *RyR2*-S2814A mice for 3 days. The CaMKII inhibitor KN-93 (KN; 2.7 mg/kg, i.p.), alone or in combination with C12-iE-DAP, was administered exclusively to WT mice for 3 days.

Echocardiography and CMR were performed to assess cardiac function. Additionally, atrial cardiomyocytes were isolated and examined by confocal microscopy to measure Ca²⁺ transients, SR Ca²⁺ load, and spontaneous sparks. Proteomic and phosphoproteomic analyses were performed using TMT18-plex labeling and Orbitrap Fusion LC-MS/MS. Further methodological details are available in the Supplementary Methods.

### Statistical analysis

Data are presented as mean ± SEM. Normality was verified by Kolmogorov-Smirnov and Shapiro-Wilk tests. Between-group comparisons were performed using unpaired Student’s *t*-tests, χ² tests, one-way or two-way ANOVA followed by Tukey’s post hoc test, or nested ANOVA as appropriate. A *P* < 0.05 was considered statistically significant. Correlations were assessed using Pearson's correlation coefficient for normally distributed data. Analyses were performed using GraphPad Prism 9 (GraphPad Software Inc., San Diego, CA, USA), Origin Pro v9.0 (OriginLab Corp., Northampton MA, USA), or SPSS22 (IBM, Armonk, NY, USA).

## Results

### NOD1 signaling is upregulated in atrial myocardium of HF patients

The study cohort comprised 81 patients undergoing valve surgery, stratified into NF (*n =* 45) and HF (*n =* 36) groups based on LVEF and NT-proBNP levels. Consistent with these criteria, HF patients showed significantly lower LVEF and elevated NT-proBNP levels compared with NF controls (Figure S2A). Baseline demographics and comorbidities were comparable between groups; however, HF patients more frequently presented with higher NYHA class III/IV and greater diuretic use (Table 1).

Echocardiographic assessment revealed marked atrial and ventricular remodeling in HF patients, characterized by increased LV volumes, elevated pulmonary pressures, and a higher E/E′ ratio (Table 1). Two-dimensional speckle-tracking demonstrated biatrial enlargement, with increased maximal and minimal LA and RA volumes accompanied by impaired reservoir, conduit, and contractile strain (Figure S2B-C). These structural and functional atrial abnormalities were associated with higher operative risk, as reflected by EuroSCORE (Figure S2D). Masson's Trichrome staining of atrial samples suggested increased collagen deposition in HF patients compared with NF controls (Figure S3A).

**Figure 1:**
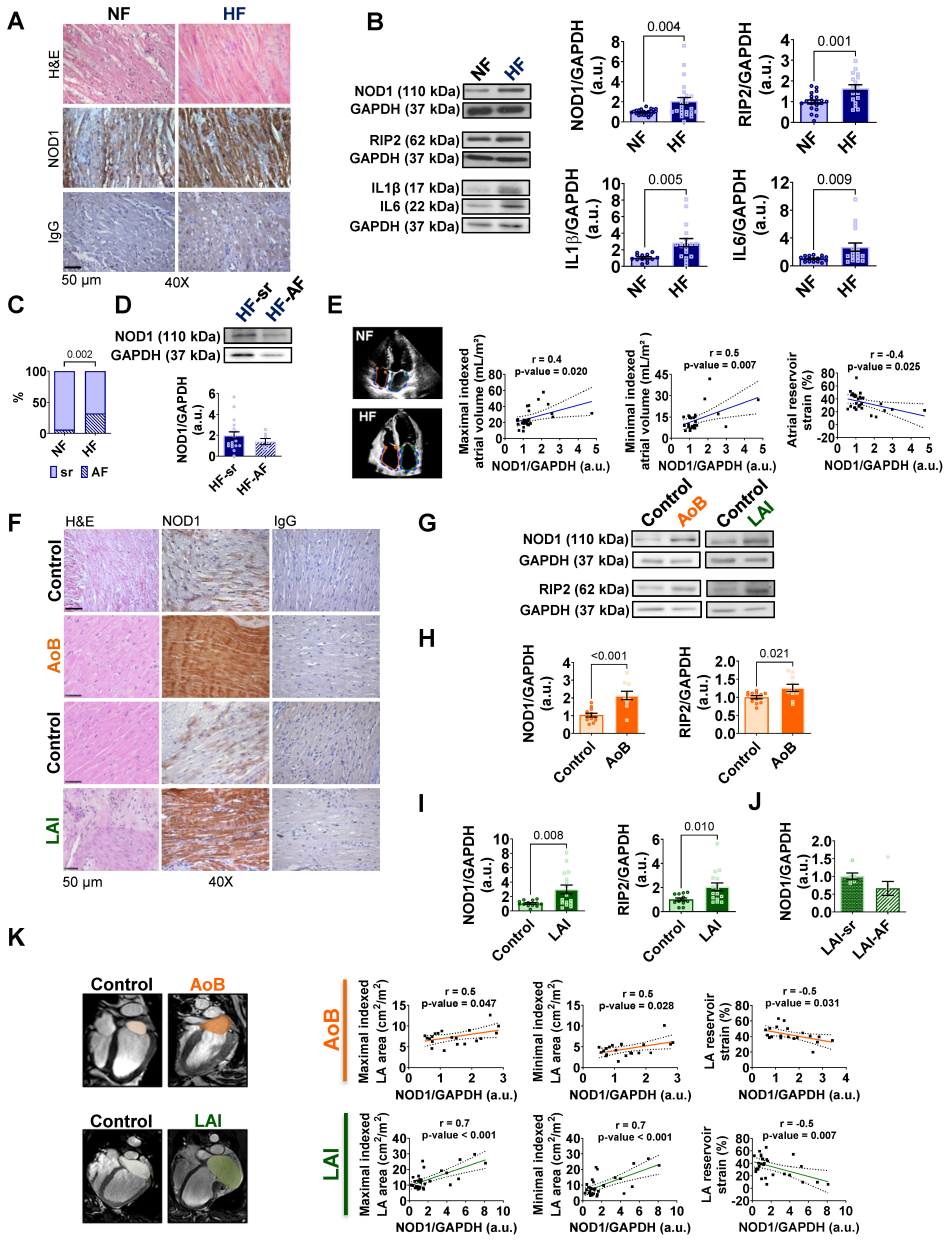
** NOD1 signaling is upregulated in atrial myocardium of patients and swine models of heart failure.** (A) Representative hematoxylin-eosin (H&E) and immunohistochemical (IHC) staining for NOD1 in atrial tissue, with isotype controls from non-failing (NF) and heart failure (HF) patients. (B) Western blot and quantification of NOD1 (NF, *n =* 24; HF, *n =* 25), its adaptor RIP2 (NF, *n =* 20; HF, *n =* 17), IL-1β (NF, *n =* 13; HF, *n =* 18), and IL6 (NF, *n =* 17; HF, *n =* 18) expression in atrial tissue, normalized to GAPDH. (C) Prevalence of AF (%) in both groups. (D) Subgroup comparison of NOD1 protein levels in HF patients in sinus rhythm (HF-SR, *n =* 18) and atrial fibrillation (HF-AF, *n =* 7). (E) Representative echocardiographic images and correlations between atrial NOD1 protein levels and maximal and minimal atrial volumes and reservoir strain (NF, *n =* 29; HF, *n =* 26). (F) Representative H&E, NOD1 IHC staining and isotype controls obtained in LA tissue from aortic banding (AoB) and left-atrial infarction (LAI) porcine models and Control animals. (G-I) Western blot and quantification of NOD1 and its adaptor RIP2 expression in left atrial (LA) tissue from (G-H) Control (*n =* 12) and AoB (*n =* 10) Yucatan minipigs and from (G-I) Control (*n =* 13) and LAI (*n =* 16) Large White Pigs, normalized to GAPDH. (J) Quantification of NOD1 protein levels in atrial tissue from a separate cohort of LAI animals (LAI-sr, *n* = 6 and LAI-AF, *n* = 6), normalized to GAPDH. (K) Representative cardiac magnetic resonance (CMR) images and correlations between LA NOD1 expression and atrial area and reservoir strain. Data are mean ± SEM; statistical analyses performed by unpaired t-test or Pearson’s correlation (*P* < 0.05 considered significant).

Immunohistochemistry revealed NOD1 localization within atrial cardiomyocytes (Figure 1A), with no evidence of macrophage, T cell, or B cell infiltration in atrial myocardial biopsies from either HF or NF patients (Figure S2E). Western blot analysis showed increased protein levels of NOD1 and its adaptor RIP2, together with elevated levels of inflammatory cytokines IL-1β and IL-6 in HF atrial tissue (Figure 1B). Although the incidence of documented AF was higher in HF patients (Figure 1C), subgroup analysis of HF stratified by rhythm status at the time of preoperative evaluation demonstrated comparable NOD1 protein levels between those in sinus rhythm and those with AF (Figure 1D), suggesting that NOD1 upregulation in the failing atrium is not contingent upon documented AF.

Next, atrial NOD1 protein levels were matched to chamber-specific echocardiographic features. NOD1 expression positively correlated with maximal and minimal atrial volumes, and inversely with atrial reservoir strain (Figure 1E). Additional correlations with atrial area, conduit and contractile strain, NT-proBNP levels, and EuroSCORE further highlighted the clinical relevance of NOD1 upregulation in failing human atria (Figure S2F-G).

Collectively, these findings establish NOD1 upregulation as a molecular hallmark of atrial remodeling in human HF, bridging innate immune activation with structural and functional atrial damage.

### NOD1 expression is increased in swine models of atrial myopathy

NOD1 expression was next assessed in two swine models of atrial myopathy: aortic banding (AoB), with preserved LVEF, and left atrial infarction (LAI), with reduced LVEF (Figure S4A). Despite the differences in ventricular phenotypes, CMR imaging confirmed significant atrial impairment in both models, including increased LA volumes and areas, reduced LA strain, and mild RA dysfunction (Figure S4B-G and Tables 2 and 3).

Immunohistochemical analysis of LA tissue from AoB and LAI swine revealed prominent NOD1 staining in atrial cardiomyocytes (Figure 1F), with no detectable macrophage, T cell, or B cell infiltration (Figure S4H-I). Western blot analyses confirmed elevated levels of NOD1 and RIP2 in LA tissue from both LAI and AoB models compared to their respective controls (Figure 1G-I). These molecular changes were accompanied by increased expression of *IL1B* and *IL6* in failing atria from both models (Figure S5A-B). Notably, in an independent LAI cohort designed for electrophysiological studies, NOD1 protein levels were comparable between animals subjected to sustained burst pacing-induced AF and those maintained under sinus rhythm conditions (Figure 1J).

Consistent with the human data, significant correlations were observed in both swine models between LA NOD1 protein levels and maximal and minimal atrial volumes, as well as atrial reservoir strain (Figure 1K). Additional associations with conduit and contractile strain further reinforced the pathophysiological relevance of NOD1 in atrial remodeling (Figure S5C-D).

Together, these results demonstrate that atrial NOD1 upregulation is a conserved feature of HF-associated atrial remodeling, observed across divergent ventricular phenotypes and irrespective of documented rhythm status.

### NOD1 deficiency prevents atrial remodeling and Ca²⁺mishandling in pressure-overload mice

To elucidate the role of NOD1 in atrial remodeling, we employed a genetic loss-of-function approach using a murine pressure-overload model of HF induced by TAC (Figure S6A). NOD1 deficiency prevented pressure overload–induced atrial remodeling. Additionally, *Nod1⁻/⁻* mice were protected from TAC-induced LV dysfunction (Table 4; Figure S6B).

**Figure 2:**
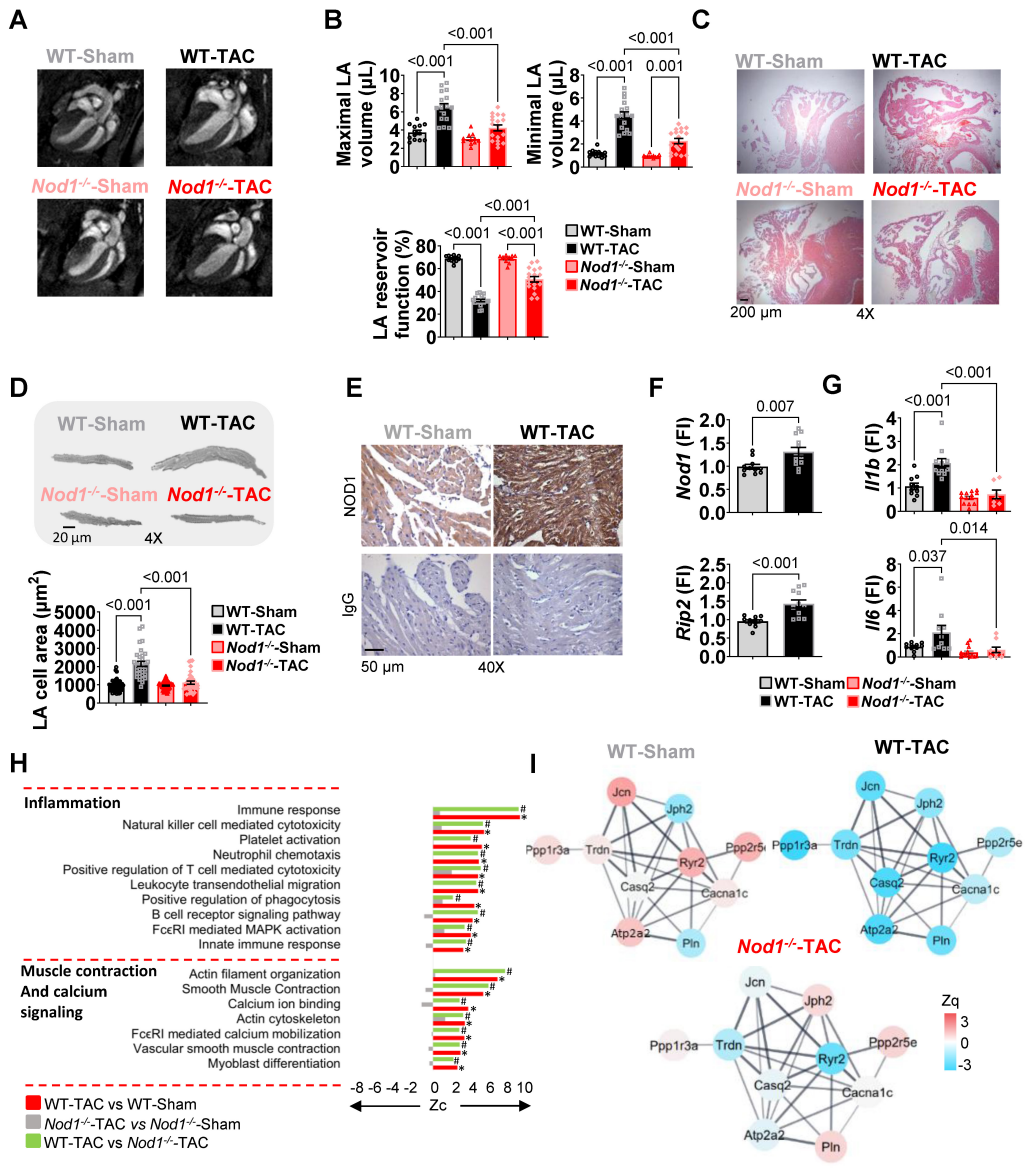
** NOD1 deficiency prevents atrial remodeling and proteomic alterations in mice subjected to pressure overload.** (A) Representative long-axis four-chamber CMR images illustrating differences in left atrial size across groups. (B) Maximal and minimal LA volumes and reservoir function obtained in WT-Sham (*n =* 13), WT-TAC (*n =* 17), *Nod1^-^/^-^*-Sham (*n =* 11) and* Nod1⁻/⁻-TAC* (*n =* 18). (C) Representative H&E staining illustrating atrial remodeling in all groups. (D) Representative images and quantification of LA cardiomyocyte cross-sectional area in WT*-*Sham (*n =* 57cells/6 mice), WT*-*TAC (*n =* 33 cells/5 mice), *Nod1^-^/^-^*-Sham (*n =* 53 cells/3 mice) and *Nod1⁻/⁻-TAC* (*n =* 35 cells/6 mice). (E) Representative IHC staining for NOD1 in LA tissue, with isotype controls. (F) LA mRNA expression of *Nod1* and *Rip2* normalized to 36B4 and relative to WT*-*Sham. (G) LA mRNA expression of* Il1b* and *Il6* normalized to 36B4 and relative to WT*-*Sham. (H) Functional enrichment analysis of murine LA tissue showing differential enrichment of inflammation- and Ca²⁺-related pathways across the indicated comparisons. Bar plots represent differential pathway enrichment for the following comparisons (n = 4 per group). Zc values represent log₂ fold-change standardized to units of standard deviation. Significant differences noted at FDR<0.05 (*WT-TAC vs. WT-Sham. #WT-TAC vs. *Nod1^⁻/⁻^*-TAC). (I) STRING protein-protein interaction network of differentially expressed Ca²⁺ handling proteins. Data are mean ± SEM; statistical analyses performed by ANOVA with Tukey’s post hoc, nested ANOVA or unpaired t-test (*P* < 0.05 considered significant).

Proteomic profiling identified 6,898 proteins, with 743 differentially expressed (FDR < 0.05) in LA tissue from WT-TAC vs. WT-Sham and 370 in WT-TAC vs. *Nod1⁻/⁻-*TAC (Figure S6E). Heatmap clustering (Figure S6F) demonstrated different molecular signatures across groups, while enrichment analysis revealed upregulation of inflammatory, Ca²⁺-handling, and extracellular matrix-related proteins, and downregulation of mitochondrial metabolism proteins in WT-TAC atria; these changes were prevented by NOD1 deficiency (Figure 2H and Figure S6G).

NOD1 deficiency conferred protection against biatrial remodeling, preventing TAC-induced increases in LA and RA weights, alterations in maximal and minimal atrial volumes, and impairment of atrial reservoir function documented in WT TAC mice (Table 4, Figure 2A-C, Figure S6C). Morphometric analyses further demonstrated that NOD1 deficiency significantly prevented TAC-induced cardiomyocyte hypertrophy (Figure 2D).

Immunohistochemistry confirmed robust NOD1 expression in LA cardiomyocytes (Figure 2E) with no macrophage, T cell, or B cell infiltration across groups (Figure S6D). qPCR analyses revealed upregulation of *Nod1* and *Rip2* transcripts, together with increased expression of *Il1b* and *Il6* in WT-TAC atria; these inflammatory markers were markedly attenuated in *Nod1⁻/⁻-*TAC atria (Figure 2F-G).

**Figure 3:**
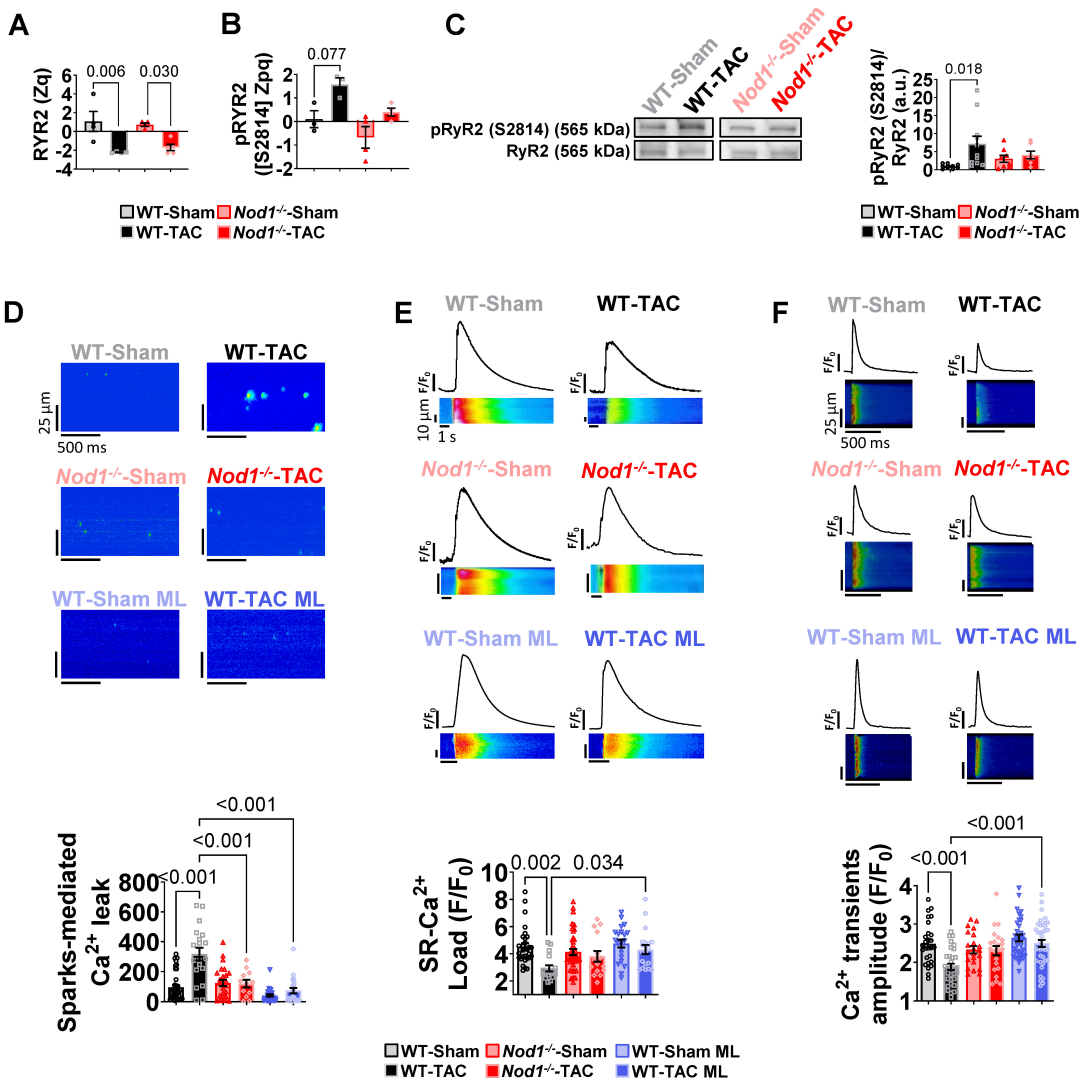
** NOD1 deficiency or pharmacological inhibition prevents TAC-induced intracellular Ca²⁺ dysregulation.** WT-Sham and WT-TAC mice were treated with the selective NOD1 inhibitor ML-130 (ML; 2 mg/kg, i.p., every other day for 4 weeks). (A-B) Quantification of RyR2 protein abundance (A; Zq values, log₂ fold-change relative to WT-Sham) and RyR2 phosphorylation at Ser2814 (B; Zpq values). Zpq values represent log₂ fold-change standardized to units of standard deviation. (C) Western blot validation of RyR2 phosphorylation at Ser2814 in LA tissue, normalized to total RyR2 (WT-Sham, *n =* 9; WT-TAC, *n =* 11; *Nod1*^-/-^-Sham, *n =* 8; *Nod1*^-/-^-TAC, *n =* 7). (D-F) Representative confocal line-scan images and quantification of (D) sparks-mediated Ca²⁺ leak indicating diastolic Ca²⁺ leak in quiescent cardiomyocytes, (E) caffeine-evoked Ca²⁺ transients reflecting sarcoplasmic reticulum (SR) Ca²⁺ load and (F) electrically evoked Ca²⁺ transients in LA cardiomyocytes from WT*-*Sham (*n =* 27-41 cells/6 mice), WT*-*TAC (*n =* 19-31 cells/5 mice), *Nod1^-/-^*-Sham (*n =* 22-42 cells/3 mice), *Nod1⁻/⁻-TAC* (*n =* 12-22 cells/6 mice), WT*-*Sham ML (*n =* 21-39 cells/3 mice), and WT*-*TAC ML (*n =* 22-40 cells/3 mice). Data are mean ± SEM; statistical analyses performed by ANOVA with Tukey’s post hoc or nested ANOVA (*P* < 0.05 considered significant).

Regarding fibrosis development, Masson's Trichrome staining suggested no changes in the collagen deposition between groups (Figure S3B); however, within the extracellular matrix proteomic signature, structural collagens Col14a1 and Col18a1, and the profibrotic matricellular proteins fibronectin (Fn1) and periostin (Postn) were all significantly elevated in WT-TAC atria and attenuated in *Nod1⁻/⁻*-TAC mice (Figure S3C). RT-PCR further confirmed transcriptional upregulation of *Tgfb1* in WT-TAC atria, which was substantially reduced in *Nod1⁻/⁻*-TAC mice (Figure S3D). These molecular alterations support the presence of active extracellular matrix remodeling, indicating early activation of profibrotic signaling, preceding overt structural fibrosis. Within the Ca²⁺-handling proteome, STRING network analysis highlighted marked differences in Ca²⁺-handling proteins between WT-TAC and *Nod1⁻/⁻*-TAC groups (Figure 2I), and quantitative proteomics confirmed that NOD1 deficiency prevented the downregulation of RyR2 and SERCA2a observed in WT-TAC atria (Figure 3A and Figure S6H).

Phosphoproteomic analysis was performed to identify post-translational regulatory changes. This analysis detected 1,451 phosphosites, with WT-TAC atria exhibiting global hyperphosphorylation that was normalized in *Nod1^⁻/⁻^-*TAC mice (Figure S6I). Notably, RyR2 emerged as a major phosphorylation hub, with phosphorylation at Ser2814 (principal CaMKII target site) markedly increased in WT-TAC but prevented in Nod1*^⁻/⁻^*-TAC atria (Tables S1, S2 and Figure 3B). Western blot analyses validated these findings, confirming that NOD1 deficiency prevented CaMKII-RyR2 Ser2814 hyperphosphorylation (Figure 3C). In parallel, levels of the PP1 regulatory subunit PP1R3A, which modulates RyR2 dephosphorylation, were reduced in WT-TAC but preserved in *Nod1^⁻/⁻^-*TAC atria (Figure S6J).

Consistent with these molecular findings, NOD1 deficiency significantly prevented TAC-induced abnormalities in atrial Ca²⁺ handling, including increased spark-mediated diastolic Ca²⁺ leak (Figure 3D), enlarged and prolonged Ca²⁺ sparks (Figure S7A), reduced SR Ca²⁺ load (Figure 3E), and decreased Ca²⁺transient amplitude (Figure 3F), while preserving SERCA2a expression (Figure S7B).

These findings identify NOD1 as a key driver of structural, molecular, and functional atrial remodeling under pressure overload in a mouse model.

### Pharmacological NOD1 inhibition reproduces the protective effects of genetic NOD1 deficiency in TAC-induced atrial myopathy

To determine whether pharmacological NOD1 inhibition could recapitulate the protective phenotype observed in *Nod1⁻/⁻-*TAC mice, we treated WT-Sham and WT-TAC mice with the selective NOD1 inhibitor ML-130 (ML; 2 mg/kg, i.p., every other day for 4 weeks). At the structural level, pharmacological NOD1 inhibition prevented the development of cardiac and atrial hypertrophy in response to pressure overload. WT-TAC mice treated with ML did not exhibit the increases in heart or atrial weight observed in untreated WT-TAC animals, and hypertrophy indices remained comparable to WT-Sham controls (Table S3). At the microscopic level, the increase in cardiomyocyte cellular area observed in WT-TAC mice was also prevented by ML treatment (Table S3). These findings closely mirror the phenotype observed in *Nod1^-/-^-*TAC mice, indicating that pharmacological inhibition of NOD1 is sufficient to prevent pressure overload–induced structural atrial remodeling.

To determine whether this structural protection was accompanied by preservation of cardiomyocyte Ca²⁺ handling, atrial cardiomyocytes were isolated for functional Ca²⁺ recordings. ML treatment had no measurable effect in WT-Sham mice, which maintained physiological Ca²⁺ handling parameters (Figures 3D-F; Figure S7A), indicating that basal NOD1 activity is low in healthy atria and that its inhibition does not disrupt physiological Ca²⁺ dynamics. In contrast, ML treatment in WT-TAC mice prevented the full spectrum of TAC-induced Ca²⁺ abnormalities, reproducing the protective effects observed in *Nod1^⁻/⁻^-*TAC mice. Specifically, pharmacological NOD1 inhibition normalized spark-mediated diastolic Ca²⁺ leak, Ca²⁺ transient amplitude, SR Ca²⁺ load, and Ca²⁺ spark properties (frequency and duration), while amplitude remained unchanged (Figures 3D-F; Figure S7A).

Together, these findings demonstrate that pharmacological NOD1 inhibition reproduces both the structural and Ca²⁺ handling protection observed in genetic NOD1 deficiency, supporting a main role for NOD1 signaling in TAC-induced atrial myopathy.

### Selective NOD1 activation induces atrial Ca²⁺ dysregulation through CaMKII-dependent RyR2-Ser2814 phosphorylation

**Figure 4:**
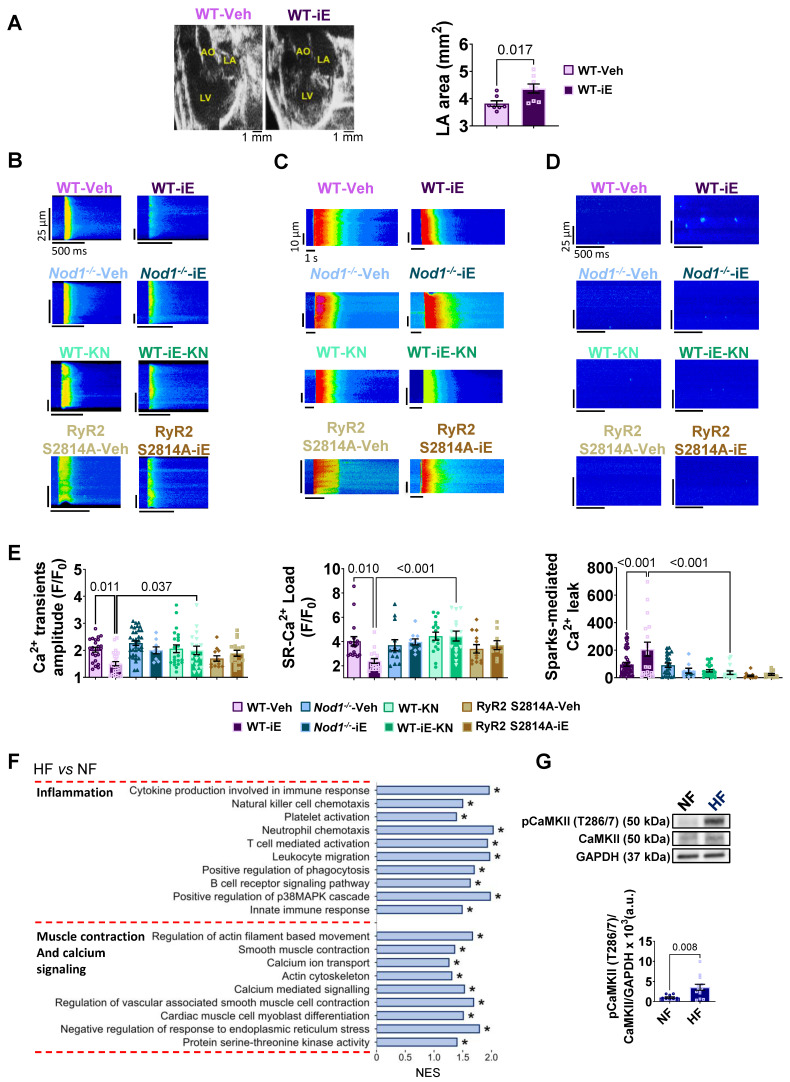
** NOD1 activation induces CaMKII-dependent Ca²⁺ mishandling in atrial cardiomyocytes. RNAseq analysis of human atrial myocardium.** WT, *Nod1^⁻/⁻^,* and *RyR2*-S2814A mice were used to assess the effects of genetic and pharmacological NOD1 modulation. (A) Representative echocardiographic images (left panel) and LA area (right panel) in WT-Vehicle (WT-Veh) and WT-C12-iE-DAP (WT-iE). (B-E) Representative confocal line-scan images and quantification of Ca²⁺ transients amplitude (C and E, left panel), SR Ca²⁺ load (C and E, central panel), and sparks-mediated diastolic Ca²⁺ leak (D and E, right panel) in WT-Veh (*n =* 17-47 cells/6 mice), WT-iE (*n =* 18-26 cells/3 mice), *Nod1*^-/-^-Veh (*n =* 13-35 cells/3 mice), *Nod1*^-/-^-iE (*n =* 9-10 cells/2 mice), WT-KN (*n =* 17-27 cells/4 mice), WT-iE-KN (*n =* 15-18 cells/3 mice), RyR2 S2814A-Veh* (n* = 12-13 cells/3 mice) and RyR2 S2814A-iE (*n* = 9-17 cells/3 mice). (F) Transcriptomic gene set enrichment analysis of human atrial tissue showing upregulation of inflammation- and Ca²⁺-related pathways in HF vs. NF patients. * indicates significantly enriched pathways at FDR < 0.05. (G) Representative Western blot (upper panel) and quantification (lower panel) of CaMKII phosphorylation at Thr-286/7 (pCaMKII T286/7), normalized to total CaMKII and GAPDH (NF, *n* = 15; HF, *n =* 17). Data are mean ± SEM; statistical analyses performed by unpaired t-test or nested ANOVA (*P* < 0.05 considered significant).

To gain a deeper understanding of the mechanistic role of NOD1 in the development of atrial remodeling, we next examined whether selective NOD1 activation is sufficient to induce atrial dysfunction and Ca²⁺ dysregulation. WT mice were treated with the selective NOD1 agonist C12-iE-DAP (iE; 3.3 mg/kg, i.p., for 3 days). Echocardiography revealed reduced LVEF and increased LA enlargement in WT mice treated with iE compared to vehicle-treated controls (Figure 4A and Figure S8A).

At the cellular level, iE administration markedly impaired systolic Ca²⁺ release, as evidenced by reduced Ca²⁺ transient amplitude (Figure 4B and E, left panel), together with decreased SR Ca²⁺ load (Figure 4C and E, central panel) in isolated LA cardiomyocytes. In parallel, iE increased diastolic Ca²⁺ leak through elevated Ca²⁺ sparks (Figure 4D and E, right panel). These alterations were absent in *Nod1⁻/⁻* mice treated with iE (Figure 4B-E), confirming the specificity of iE for NOD1-dependent mediation of atrial Ca²⁺ dysregulation. To identify the downstream effector mechanism, we examined RyR2 phosphorylation status. iE-treated WT mice exhibited a selective increase in CaMKII-dependent RyR2 phosphorylation at Ser2814 (Figure S8B).

To determine whether CaMKII signaling mediates the atrial Ca²⁺ mishandling induced by NOD1 activation, WT mice were co-treated with iE and the CaMKII inhibitor KN-93 (KN; 2.7 mg/kg, i.p., for 3 days). KN treatment completely prevented iE-induced Ca²⁺ handling impairment, normalizing Ca²⁺ transient amplitude, SR Ca²⁺ load, and Ca²⁺ spark-mediated diastolic Ca²⁺ leak (Figure 4B-E).

Genetic validation of RyR2-Ser2814 phosphorylation as the critical mechanistic target was obtained using *RyR2*-S2814A mice, in which serine 2814 is mutated to alanine preventing phosphorylation at this site. In this model, iE failed to induce atrial Ca²⁺ handling abnormalities (Figure 4B-E), corroborating the effects observed under pharmacological CaMKII inhibition. Accordingly, atrial cardiomyocytes from* RyR2*-S2814A mice treated with iE exhibited Ca²⁺ transient amplitude, SR Ca²⁺ load, and Ca²⁺ spark-mediated diastolic Ca²⁺ leak comparable to those observed in vehicle-treated *RyR2*-S2814A controls. Together, these data identify CaMKII-dependent RyR2-Ser2814 phosphorylation as a central mechanistic node linking NOD1 activation to atrial Ca²⁺ dysregulation.

To determine whether acute NOD1 activation was sufficient to increase AF susceptibility, transesophageal burst pacing was performed in WT mice treated with iE or vehicle. AF was induced in 2 of 7 iE-treated animals compared with 1 of 7 vehicle-treated controls, with no significant differences in AF inducibility between groups (Figure S8C). These findings indicate that, while acute NOD1 activation is sufficient to reproduce cardiomyocyte Ca²⁺ dysregulation associated with triggered activity, the short-term pharmacological protocol does not lead to a detectable increase in AF inducibility under these experimental conditions.

### Atrial myocardium from patients with HF displays CaMKII hyperactivation

To evaluate the translational relevance of these experimental findings, we performed transcriptomic profiling of atrial myocardium from HF and NF patients. RNA sequencing identified 15,508 expressed genes, with 463 genes upregulated and 184 downregulated in HF atrial tissue (FDR < 0.05). Clustering of the top 70 differentially expressed genes clearly distinguished HF from NF samples (Figure S9A and Table S4).

Consistent with the molecular signatures identified in our murine models, gene set enrichment analysis revealed significant enrichment of pathways associated with inflammation and muscle contraction/Ca²⁺ signaling in atrial myocardium from HF patients compared with NF samples (Figure 4F). Pathways related to extracellular matrix organization and cell–matrix interactions were also enriched (Figure S9B), consistent with structural remodeling in the human atrium, whereas no significant enrichment was observed for mitochondrial metabolism or energy-related pathways (Figure S9B).

At the protein level, Western blot analysis demonstrated increased CaMKII phosphorylation (Thr-286/7) in HF atrial myocardium relative to NF controls (Figure 4G). These results establish CaMKII hyperactivation as a conserved molecular feature of human HF-associated atrial myopathy.

## Discussion

HF imposes a significant clinical burden and is associated with high mortality, underscoring the need to better define the molecular mechanisms that drive disease progression. Growing evidence implicates sustained activation of innate immune receptors as a key contributor to maladaptive inflammatory signaling underlying HF-associated atrial myopathy 20. Among NLRs, the NLRP3 inflammasome has been extensively studied for its role in cardiac inflammation and remodeling 14,16,20-22. In contrast, the involvement of NOD1, another NLR family member, in clinical and experimental atrial myopathy remains underexplored.

Our study demonstrates marked NOD1 upregulation in atrial myocardium from HF patients and two porcine models with divergent ventricular phenotypes (AoB with preserved LVEF and LAI with reduced LVEF), with a consistent expression pattern observed across the study cohorts irrespective of documented rhythm status, suggesting that NOD1 upregulation reflects the structural and hemodynamic burden of HF-associated atrial remodeling rather than the underlying rhythm phenotype.

Immunohistochemical analysis revealed no macrophage, T cell, or B cell infiltration in atrial tissue across all species examined, positioning cardiomyocytes as the primary NOD1-expressing cellular compartment. This pattern was conserved across humans, porcine, and murine models, supporting a primary role for cardiomyocytes in NOD1-mediated atrial remodeling, rather than secondary immune cell recruitment.

Elevated NOD1 expression in atrial myocardium from HF patients and porcine models strongly correlated with structural and functional markers of atrial remodeling, including atrial enlargement and impaired atrial strain. Atrial strain has emerged as a sensitive and promising marker of atrial dysfunction, improving diagnostic precision and independently predicting mortality in HF, irrespective of underlying etiology 23,24. The association between NOD1 expression and both structural and functional atrial impairment highlights its potential as a clinically relevant molecular determinant of atrial pathology in HF.

AF represents the most common arrhythmia in HF and significantly increases morbidity and mortality worldwide 25. Although AF incidence was higher among HF patients in our cohort, atrial NOD1 protein levels were comparable between HF patients with and without documented AF at the time of preoperative evaluation. This observation was mirrored in the porcine LAI model, where NOD1 expression remained similarly elevated in both LAI-AF and LAI-sr animals, suggesting that NOD1 upregulation is associated with the underlying structural atrial remodeling rather than arrhythmia *per se*. These findings are consistent with prior evidence demonstrating that atrial structural and molecular remodeling can precede AF onset and progress independently of arrhythmia burden in patients with HF 4.

The combined use of genetic loss-of-function and pharmacological inhibition approaches provides strong evidence supporting a role for NOD1 signaling in HF-associated atrial myopathy. *Nod1^⁻/⁻^* mice subjected to TAC exhibited markedly attenuated atrial enlargement and preserved atrial function compared with WT-TAC controls, supporting a role for NOD1 signaling as a key contributor to atrial remodeling under pressure overload. Notably, the absence of immune cell infiltration (CD68⁺ macrophages, CD3⁺ T cells, and CD19⁺ B cells) in atria from WT-TAC and *Nod1^⁻/⁻^*-TAC mice supports a cardiomyocyte-intrinsic mechanism, consistent with the established role of PRRs as cell-autonomous inflammatory sensors within the myocardium 21. In parallel, *Nod1*⁻/⁻-TAC mice displayed attenuated LV dysfunction, suggesting that part of the observed atrial protection may be secondary to improved ventricular performance. Importantly, complementary gain-of-function experiments *in vivo* using the selective NOD1 agonist C12-iE-DAP induced atrial Ca²⁺ dysregulation in otherwise healthy mice, in the absence of overt HF or LV impairment, as previously reported 17. Together, these findings support a cardiomyocyte-centered role for NOD1 in atrial Ca²⁺ handling, independent of hemodynamic confounders.

In parallel, structural remodeling was also evident at the level of the extracellular matrix. In human atrial samples, transcriptomic profiling revealed enrichment of pathways related to extracellular matrix organization and cell–matrix interactions. In the murine model, the analyses demonstrated that NOD1 deficiency in TAC mice prevented the dysregulation of extracellular matrix component expression, including fibrillar collagens, fibronectin, and periostin compared with the WT-TAC group. These findings indicate that NOD1 might contribute to structural remodeling in atrial myopathy.

While cardiomyocytes represent a major site of NOD1 expression in the atrium, NOD1 is also expressed in cardiac fibroblasts and endothelial cells, suggesting that NOD1 signaling in non-cardiomyocyte populations may further modulate atrial remodeling. The present study, however, primarily supports a cardiomyocyte-centered mechanism underlying NOD1-mediated atrial dysfunction, and the contribution of non-myocyte cell populations remains to be defined.

Beyond these structural alterations, proteomic, and molecular analyses revealed that NOD1 deficiency preserved the expression of key Ca²⁺-handling proteins, including SERCA2a and RyR2, in TAC-subjected mice. This preservation resulted in normalized SR Ca²⁺ reuptake, Ca²⁺ transient amplitude, and SR Ca²⁺ load, translating into improved atrial Ca²⁺ cycling and contractile performance. Importantly, pharmacological NOD1 inhibition using ML-130 reproduced the protective effects observed in *Nod1⁻/⁻* mice, preventing both TAC-induced Ca²⁺ mishandling and atrial hypertrophy, thereby strengthening the implication of NOD1 signaling in atrial remodeling.

At the post-translational level, NOD1 deficiency further prevented pathological signaling remodeling 26-29. Phosphoproteomic profiling revealed global hyperphosphorylation in WT-TAC atria, with RyR2 emerging as a major phosphorylation hub. Critically, NOD1 deficiency prevented CaMKII-mediated hyperphosphorylation of RyR2 at Ser2814, the principal CaMKII target site. This selective prevention of CaMKII-dependent RyR2 phosphorylation markedly reduced diastolic Ca²⁺ leak, a critical driver of atrial dysfunction and enlargement 30.

RyR2 phosphorylation is tightly regulated by a balance of kinases and phosphatases 26-29. Consistent with increased RyR2 phosphorylation in WT-TAC atria, we observed reduced expression of the phosphatase regulatory subunit PP1R3A, whose downregulation has been associated with increased RyR2 phosphorylation and SR Ca²⁺ leak 28. NOD1 deficiency prevented PP1R3A downregulation, reinforcing the protective role of NOD1 deficiency in HF-associated atrial myopathy.

Pathological diastolic SR Ca²⁺ leak in WT-TAC atrial myocytes contributes to atrial dysfunction and enlargement 21,31. Beyond excitation-contraction coupling, aberrant Ca²⁺ signaling activates Ca²⁺-sensitive transcriptional programs, including CaMKII-dependent pathways that promote maladaptive remodeling 32. Accordingly, the absence of atrial hypertrophy in *Nod1^⁻/⁻^*-TAC mice is consistent with normalized Ca²⁺ handling and reduced CaMKII activation. These findings align with prior evidence showing that pathological SR Ca²⁺ leak accelerates maladaptive remodeling and HF progression under pressure overload 33.

The pathogenic role of NOD1 in atrial Ca²⁺ dysregulation was further validated through gain-of-function studies. Acute administration of the selective NOD1 agonist C12-iE-DAP in WT mice induced Ca²⁺-handling abnormalities closely resembling those observed in the TAC mouse model, including impaired systolic Ca²⁺ release, reduced SR Ca²⁺ load, and increased RyR2-mediated diastolic Ca²⁺ leak. These effects were abolished by pharmacological CaMKII inhibition with KN-93, establishing strict dependence on CaMKII activation. Importantly, *RyR2*-S2814A mice (phosphorylation-deficient at Ser2814) were protected from NOD1-driven Ca²⁺ mishandling, identifying CaMKII-dependent RyR2-Ser2814 phosphorylation as the critical mechanistic node linking NOD1 activation to atrial Ca²⁺ dysregulation. Importantly, AF inducibility was not significantly increased following acute NOD1 activation, in line with the data obtained from patients and the LAI-AF swine model.

These findings uncover the NOD1–CaMKII–RyR2 axis as a cardiomyocyte-centered pathway linking innate immune activation to atrial Ca²⁺ mishandling, in line with recent reports implicating IL-1β-NLRP3 signaling in CaMKII-dependent RyR2 hyperphosphorylation and pathological Ca²⁺ leak in human atrial cardiomyocytes 14. The translational relevance of this mechanistic framework is directly supported by molecular analyses of human HF atrial myocardium, which revealed enrichment of inflammatory and Ca²⁺ signaling pathways, together with increased CaMKII phosphorylation at Thr-286/287, indicating that CaMKII hyperactivation is a conserved feature of the failing human atrium and that the NOD1–CaMKII–RyR2 axis identified in experimental models operates in the clinical setting.

Collectively, these findings position NOD1 as a molecular mediator of HF–associated atrial myopathy, with potential applications in both patient stratification and mechanism-based intervention. From a diagnostic standpoint, atrial NOD1 expression correlated with established indices of atrial disease severity across divergent ventricular phenotypes and irrespective of documented rhythm status, supporting NOD1 pathway activation as a molecular signature of atrial remodeling burden that may refine patient stratification beyond conventional clinical parameters. From a therapeutic standpoint, both genetic deletion and pharmacological inhibition of NOD1 attenuated atrial Ca²⁺ dysregulation and structural remodeling, providing convergent preclinical evidence for NOD1 targeted intervention. Mechanistically, the identification of CaMKII-dependent RyR2-Ser2814 phosphorylation as the critical downstream effector defines a therapeutically actionable axis, positioning the NOD1–CaMKII–RyR2 pathway as a tractable multi-level target for intervention. Together, these findings establish a framework for the development of clinically applicable strategies to both monitor and modulate NOD1 pathway activity in HF-associated atrial myopathy.

### Limitations

Several limitations of the present study warrant consideration. Rhythm classification in the human cohort was based on standard 12-lead electrocardiography and systematic review of clinical records, without continuous ambulatory monitoring; accordingly, a proportion of patients categorized as sinus rhythm may have had undetected paroxysmal AF. An analogous consideration applies to the porcine LAI model, in which continuous rhythm monitoring was available in the AF subgroup through implanted devices, but not in the sinus rhythm subgroup; therefore, low-burden or transient atrial arrhythmias cannot be fully excluded in these animals. Future studies incorporating prolonged rhythm monitoring will enable more precise characterization of the relationship between NOD1 expression and AF burden. Although the present study primarily supports a cardiomyocyte-centered mechanism, the contribution of non-myocyte cell populations, including fibroblasts and endothelial cells, to NOD1-mediated atrial remodeling remains to be defined and warrants further investigation.

## Conclusions

This study identifies NOD1 as a previously unrecognized contributor to HF-associated atrial myopathy, acting through a cardiomyocyte-centered inflammatory pathway that converges on CaMKII-dependent RyR2 hyperphosphorylation and pathological Ca²⁺ mishandling. The correlation of atrial NOD1 expression with structural and functional indices of disease severity, together with the preclinical efficacy of NOD1 blockade, establishes the NOD1–CaMKII–RyR2 axis as both a molecular indicator of atrial remodeling burden and a mechanism-based therapeutic target in HF-associated atrial myopathy.

## Supplementary Material

Supplementary methods, figures and tables.

## Figures and Tables

**Table 1:** ** Baseline characteristics of non-failing (NF) and heart failure (HF) patients.** Data are mean ± SEM or %; statistical analyses performed by unpaired t-test or χ2 test (*P* < 0.05 considered significant).

	NF n = 42	HF n = 36	*P* value
**Demographics**			
Age (years)	61.83 ± 1.87	65.58 ± 1.62	0.140
Female (%)	30.95	25.00	0.620
**Clinical profile**			
Body mass index (kg/m^2^)	27.63 ± 0.69	26.68 ± 0.79	0.288
Diabetes mellitus (%)	14.29	27.78	0.168
Hypertension (%)	57.14	61.11	0.819
Dyslipidemia (%)	57.14	52.78	0.820
**Functional classification**			
NYHA III/IV (%)	0	25.00	< 0.001
**Medication use**			
Diuretics (%)	21.62	65.63	< 0.001
Angiotensin-converting enzyme inhibitors (%)	40.54	34.38	0.628
Angiotensin receptor blockers (%)	21.62	25.00	0.781
Beta-adrenergic blockers (%)	38.24	62.50	0.084
**Laboratory measurements**			
Creatinine (mg/dL)	0.89 ± 0.03	1.02 ± 0.07	0.066
Hemoglobin (g/dL)	14.33 ± 0.20	13.97 ± 0.23	0.245
C-reactive protein (mg/L)	2.73 ± 0.37	3.76 ± 0.76	0.209
Creatinine clearance (mL/min)	82.05 ± 1.70	73.16 ± 3.03	0.010
**Echocardiographic parameters**			
Indexed LVEDV (mL/m^2^)	49.90 ± 3.02	65.32 ± 6.92	0.035
Indexed LVESV (mL/m^2^)	17.65 ± 1.51	35.98 ± 4.09	< 0.001
PASP (mmHg)	23.25 ± 2.93	34.56 ± 2.83	0.030
Maximum TRPG (mmHg)	10.75 ± 2.65	25.73 ± 2.62	0.005
Lateral E/E′ ratio	10.28 ± 1.08	16.71 ± 3.40	0.043

E/E′, ratio of early mitral inflow velocity (E) to early diastolic mitral annular velocity (E′) by tissue Doppler; LVEDV, left ventricular end-diastolic volume; LVESV, left ventricular end-systolic volume; NYHA, New York Heart Association; PASP, pulmonary artery systolic pressure; TRPG, tricuspid regurgitation peak gradient.

**Table 2:** ** Cardiac magnetic resonance-derived ventricular parameters in Control and AoB Yucatan minipigs.** Data are mean ± SEM; statistical analyses performed by unpaired t-test (*P* < 0.05 considered significant).

	Control n = 17	AoB n = 13	*P* value
**Body measurements**			
Body weight (kg)	71.24 ± 3.05	84.88 ± 9.82	0.003
Body mass index (kg/m^2^)	1.439 ± 0.04	1.611 ± 0.03	0.003
**Cardiac magnetic resonance parameters**			
Heart rate (bpm)	70.18 ± 4.33	79.00 ± 7.06	0.273
LV mass (g)	63.70 ± 3.65	95.05 ± 7.62	0.001
Indexed LV mass (g/m^2^)	53.21 ± 3.83	58.78 ± 4.15	0.335
LV wall thickness (mm)	10.43 ± 0.73	10.98 ± 0.76	0.613
Indexed LV wall thickness (mm/m^2^)	7.38 ± 0.62	6.96 ± 0.51	0.609
LVED diameter (mm)	44.15 ± 1.45	46.42 ± 1.50	0.290
Indexed LVED diameter (mm/m^2^)	31.04 ± 1.28	29.33 ± 0.93	0.278
LVES diameter (mm)	29.90 ± 1.52	27.71 ± 1.60	0.333
Indexed LVES diameter (mm/m^2^)	20.71 ± 1.17	18.60 ± 0.69	0.111
LVEDV (mL)	96.18 ± 7.09	99.94 ± 10.11	0.756
Indexed LVEDV (mL/m²)	66.74 ± 4.27	61.45 ± 5.44	0.444
LVESV (mL)	36.59 ± 3.81	22.84 ± 2.83	0.014
Indexed LVESV (mL/m²)	25.40 ± 2.37	14.36 ± 1.76	0.002

LVED, left ventricular end-diastolic; LVEDV, left ventricular end-diastolic volume; LVES, left ventricular end-systolic; LVESV, left ventricular end-systolic volume.

**Table 3:** ** Cardiac magnetic resonance-derived ventricular parameters in Control and LAI Large White pigs.** Data are mean ± SEM; statistical analyses performed by unpaired t-test (*P* < 0.05 considered significant).

	Control n = 14	LAI n = 21	*P* value
**Body measurements**			
Body weight (kg)	66.36 ± 3.93	59.45 ± 2.11	0.102
Body mass index (kg/m^2^)	1.37 ± 0.05	1.284 ± 0.03	0.121
**Cardiac magnetic resonance parameters**			
Heart rate (bpm)	73.71 ± 3.49	69.05 ± 4.08	0.424
LV mass (g)	70.04 ± 4.34	82.13 ± 3.25	0.031
Indexed LV mass (g/m^2^)	53.01 ± 2.8	64.05 ± 2.13	0.002
LV wall thickness (mm)	7.52 ± 0.35	9.88 ± 0.87	0.046
Indexed LV wall thickness (mm/m^2^)	5.56 ± 0.33	7.88 ± 0.78	0.031
LVED diameter (mm)	51.28 ± 1.86	64.37 ± 1.82	< 0.001
Indexed LVED diameter (mm/m^2^)	37.59 ± 1.09	50.66 ± 1.80	< 0.001
LVES diameter (mm)	35.29 ± 1.23	50.17 ± 1.63	< 0.001
Indexed LVES diameter (mm/m^2^)	28.15 ± 1.81	40.41 ± 2.38	< 0.001
LVEDV (mL)	121.70 ± 8.53	179.80 ± 8.09	< 0.001
Indexed LVEDV (mL/m²)	87.65 ± 4.16	140.20 ± 5.47	< 0.001
LVESV (mL)	45.86 ± 4.01	104.00 ± 6.23	< 0.001
Indexed LVESV (mL/m²)	32.91 ± 2.30	81.13 ± 4.42	< 0.001

LVED, left ventricular end-diastolic; LVEDV, left ventricular end-diastolic volume; LVES, left ventricular end-systolic; LVESV, left ventricular end-systolic volume.

**Table 4:** ** Macroscopic and cardiac magnetic resonance-derived ventricular parameters in WT and *Nod1^-/-^*-Sham and TAC mice.** Data are mean ± SEM; statistical analyses performed by ANOVA with Tukey’s post hoc (*P* < 0.05 considered significant). * *P* < 0.05, ** *P* < 0.01, *** *P* < 0.001 *vs*. WT-Sham; ^#^ *P* < 0.05, ^##^ *P* < 0.01, ^###^ *P* < 0.001 *vs*. WT-TAC; ^&&&^ *P* < 0.001 *vs*. *Nod1^-/-^-*Sham.

	WT-Sham n = 15	WT-TAC n = 17	*Nod1*^-/-^-Sham n = 16	*Nod1*^-/-^-TAC n = 18
**Body parameters**				
BW (g)	27.88 ± 0.51	28.88 ± 0.58	27.82 ± 0.44	28.08 ± 0.68
TL (mm)	17.18 ± 0.10	17.25 ± 0.11	17.30 ± 0.10	17.10 ± 0.12
HW (mg)	178.3 ± 4.22	313.30 ± 9.48***	174.30 ± 3.54^###^	221.50 ± 8.49***^, ###, &&&^
LAW (mg)	6.393 ± 0.28	21.76 ± 0.92***	5.33 ± 0.26^###^	7.51 ± 0.39^###^
RAW (mg)	6.47 ± 0.43	10.51 ± 0.70***	5.54 ± 0.26^###^	5.92 ± 0.39^###^
HW/BW (mg/g)	6.40 ± 0.14	10.94 ± 0.43***	6.28 ± 0.12^###^	7.89 ± 0.24**^, ###, &&&^
LAW/BW (mg/g)	0.23 ± 0.01	0.76 ± 0.04***	0.19 ± 0.01^###^	0.27 ± 0.03^###^
RAW/BW (mg/g)	0.23 ± 0.01	0.37 ± 0.03***	0.20 ± 0.01^###^	0.21 ± 0.01^###^
HW/TL (mg/mm)	10.38 ± 0.23	18.20 ± 0.61***	10.08 ± 0.20^###^	12.95 ± 0.48***^, ###, &&&^
LAW/TL (mg/mm)	0.37 ± 0.01	1.27 ± 0.06***	0.31 ± 0.02^###^	0.44 ± 0.04^###^
RAW/TL (mg/mm)	0.38 ± 0.02	0.62 ± 0.04***	0.32 ± 0.01^###^	0.35 ± 0.02^###^
**Cardiac magnetic resonance parameters**				
Heart rate (bpm)	439.3 ± 13.93	465.6 ± 12.93	430.9 ± 17.93	464.5 ± 30.95
LV mass (mg)	76.08 ± 1.69	104.50 ± 4.59***	66.94 ± 2.99^###^	86.11 ± 3.31^##, &&&^
LV wall thickness (mm)	0.89 ± 0.02	1.27 ± 0.02***	0.97 ± 0.02^###^	1.04 ± 0.02***^,###^
LVED diameter (mm)	3.86 ± 0.07	4.54 ± 0.06***	3.94 ± 0.10^###^	4.06 ± 0.10^###^
LVES diameter (mm)	2.39 ± 0.05	3.39 ± 0.10***	2.60 ± 0.08^###^	2.94 ± 0.11*^**,##^
LVEDV (μL)	35.80 ± 1.28	50.23 ± 2.64**	33.41 ± 2.34^###^	39.92 ± 2.92^#^
LVESV (μL)	10.63 ± 0.95	25.58 ± 2.59***	12.19 ± 1.17^###^	18.33 ± 2.27*^, #^

BW, body weight; HW, heart weight; LAW, left atrial weight; LVED, left ventricular end-diastolic; LVEDV, left ventricular end-diastolic volume; LVES, left ventricular end-systolic; LVESV, left ventricular end-systolic volume; RAW, right atrial weight; TL, tibia length.

## Data Availability

The raw proteomics data have been deposited in the jPOST repository (accession: **JPST003217,** key **6433)**. Raw data from RNA-Seq data have been deposited in the European Nucleotide Archive (ENA, EMBL-EBI) under accession number **PRJEB107793** and will be released upon publication. Additional data supporting the findings of this study are available from the corresponding author upon reasonable request.

## Abbreviations

AF: atrial fibrillation
AoB: aortic banding
BW: body weight
CaMKII: Ca²⁺/calmodulin-dependent protein kinase II
CMR: cardiac magnetic resonance
DAMPs: damage-associated molecular patterns
E/E′: ratio of early mitral inflow velocity to early mitral annular velocity
FDR: false discovery rate
GAPDH: glyceraldehyde-3-phosphate dehydrogenase
HF: heart failure
HFmrEF: heart failure with mildly reduced ejection fraction
HFrEF: heart failure with reduced ejection fraction
HW: heart weight
IHC: immunohistochemistry
IL1B: interleukin 1 beta
IL6: interleukin 6
LA: left atrium
LAI: left atrial infarction
LAW: left atrial weight
LV: left ventricle
LVEF: left ventricular ejection fraction
LVEDV: left ventricular end-diastolic volume
LVESV: left ventricular end-systolic volume
NF: non-failing
NLRs: NOD-like receptors
NOD1: nucleotide-binding oligomerization domain-containing protein 1
NT-proBNP: N-terminal pro-B-type natriuretic peptide
NYHA: New York Heart Association
PAMPs: pathogen-associated molecular patterns
PASP: pulmonary artery systolic pressure
PP1: protein phosphatase 1
PRRs: pattern recognition receptors
RA: right atrium
RAW: right atrial weight
RIP2: receptor-interacting serine/threonine-protein kinase 2
RyR2: ryanodine receptor 2
SERCA2a: sarco/endoplasmic reticulum Ca²⁺-ATPase 2a
SR: sarcoplasmic reticulum
TAC: transverse aortic constriction
TL: tibia length
TRPG: tricuspid regurgitation peak gradient
WT: wild-type
