# Supplementary material for: NOD1 is a key mediator of atrial myopathy in heart failure
Source: Theranostics. 2026 Jun 4;16(13):7244–58. doi: 10.7150/thno.134756 (PMC13294985; doi:10.7150/thno.134756)
Supplement: Supplementary file 1 — Supplementary methods, figures and tables. [file thnov16p7244s1.pdf]

Supplementary Methods

Human Studies

Patient Selection and Clinical Assessment

Atrial myocardial samples were obtained from non-failing (NF) and heart failure (HF) patients undergoing valve replacement or repair at La Paz University Hospital (Madrid, Spain). Right and left atrial (LA) appendages were excised intraoperatively, immediately snap-frozen in liquid nitrogen, and stored at −80 °C until analysis. Peripheral blood was collected in EDTA tubes, centrifuged at 2,500 rpm for 10 min at 4°C, and plasma was stored at −80 °C. Biochemical parameters (Table 1), including NT-proBNP levels (Figure S1A), were quantified at the Clinical Analytics Service of La Paz University Hospital using a chemiluminescence assay (Atellica Solution IM 1600, Siemens Healthineers, Erlangen, Germany). All patients provided written informed consent, and the study was approved by the Human Ethics Committee of La Paz University Hospital (CEIm: 1729) and conducted in accordance with the Declaration of Helsinki.

Echocardiographic Analysis

Comprehensive transthoracic echocardiography was performed preoperatively using a Philips iE33 system (Philips Healthcare, Andover, MA, USA) by a certified sonographer blinded to clinical information. Offline analyses employed QLAB 13 software following ASE/EACVI guidelines [1]. Left ventricular ejection fraction (LVEF) was calculated by the modified biplane Simpson's rule. LA and right atrial (RA) volumes were measured using the biplane disk method and indexed to body surface area. Two-dimensional speckle-tracking echocardiography on apical four-chamber view loops (60-80 Hz) was used to assess phasic atrial strain (reservoir, conduit, and contractile phases). The atrial strain parameters were derived from QRS-triggered strain curves [2]. Three-dimensional echocardiography was performed when feasible for validation.

RNA Sequencing and Bioinformatics

Total RNA was extracted from atrial tissue using TRIzol (Thermo Fisher Scientific, Waltham, MA, USA). RNA integrity was assessed using an Agilent Bioanalyzer 2100 (Agilent Technologies, Santa Clara, CA, USA), and only samples with an RNA Integrity Number (RIN) ≥7.0 were included. RNA sequencing was performed on atrial samples from NF (n = 3) and HF (n = 3) at BGI Genomics (Shenzhen, China) using DNBseq technology (MGISEQ-2000RS), generating more than 30 million 100-bp paired-end reads per sample.

Raw reads were trimmed and low-quality reads were removed using Skewer v0.2.2 [3]. Clean reads were aligned to the human reference genome (GRCh38) using STAR [4], and gene- and isoform-level expression was quantified with RSEM (RNA-Seq by Expectation-Maximization) [5,6]. Differential gene expression analysis was performed using DESeq2 (v1.36.0) [7], applying thresholds of $|\log_2$ fold change$|$ >1 and adjusted p-value (FDR) <0.05.

Gene Ontology and Kyoto Encyclopedia of Genes and Genomes (KEGG) pathway enrichment analyses were conducted using gene set enrichment analysis (GSEA) implemented in the clusterProfiler package, with false discovery rate correction (FDR <0.05). Although the number of human atrial samples was limited by tissue availability, this exploratory transcriptomic analysis was designed to identify conserved molecular pathways subsequently validated in murine models.

Swine models

All swine procedures conformed to the Spanish Animal Care and Use Committee regulations (2013/175; EU Directive 2010/63/EU) and were authorized by the General Direction of Agriculture and Environment (PROEX 103/19).

General Surgical Procedures

Animals were anesthetized with intramuscular ketamine (20 mg/kg), xylazine (2 mg/kg), and midazolam (0.5 mg/kg), and were maintained by continuous intravenous infusion of ketamine

(2 mg/kg/h), xylazine (0.2 mg/kg/h), and midazolam (0.2 mg/kg/h). Mechanical ventilation was set to 30% oxygen with tidal volumes of 8–10 mL/kg and 15 breaths/min. Femoral arterial and venous access were established via the Seldinger technique. Intraoperative anticoagulation included unfractionated heparin (300 IU/kg bolus) and amiodarone (300 mg/h infusion). Postoperative analgesia was administered using intramuscular buprenorphine (0.01 mg/kg). Pigs were euthanized with intravenous sodium pentobarbital (40 mg/kg) at protocol-defined time points. LA tissue was either immediately processed for histological analysis or snap-frozen and stored at -80 °C for subsequent biochemical and molecular studies.

Aortic Banding (AoB) Model

Supravalvular aortic stenosis was induced in 12-month-old male Yucatan minipigs (*n* = 13) via right lateral thoracotomy under general anesthesia (isoflurane 2-3%, 1 L/min O_2) [8]. A 10-mm Dacron band was placed 3 cm above the aortic valve, tightened to 70% of the aortic perimeter (measured intraoperatively with calipers), and secured with a single 4-0 Prolene stitch penetrating the aortic adventitia-media layer on one side. This created a slightly elliptical stenosis, increasing in severity with animal growth, modeling progressive supravalvular aortic stenosis and eccentric flow. No palpable thrill was detected, ensuring non-obstructive banding. Postoperative analgesia was administered via intramuscular buprenorphine (0.01 mg/kg) before recovery. Sham-operated controls (*n* = 17, same age and sex) underwent identical procedures without band tightening. Pigs were euthanized 32 weeks post-surgery with sodium pentobarbital (40 mg/kg, intravenous). All animals underwent longitudinal imaging assessment. Molecular analyses were performed in a subset of animals, based on experimental allocation and tissue availability.

Left Atrial Infarction (LAI) Model

Left atrial injury was induced in 3-month-old male Large White piglets (*n* = 21) by percutaneous coil occlusion of the LA branch of the left circumflex coronary artery under

fluoroscopic guidance (isoflurane 2–3%, 1 L/min O_2). Sham-operated controls (*n* = 14, same age and sex) underwent catheterization without occlusion. Animals were euthanized 8 weeks post-surgery. All animals underwent longitudinal imaging assessment. Molecular analyses were performed in a subset of animals, based on experimental allocation and tissue availability.

Intracardiac electrophysiology in LAI animals

A separate cohort of LAI animals (*n* = 12) was used for intracardiac electrophysiological studies. Eight weeks after surgery, animals were allocated to either the LAI AF group (n = 6) or the LAI sr group (*n* = 6) to enable comparison under matched follow-up conditions. Animals assigned to the LAI AF group underwent dual-chamber pacemaker implantation (Accent DR-RF, Abbott, Chicago, IL, USA). Atrial and ventricular leads (Tendril STS Pacing Lead, Abbott Park, IL, USA) were inserted via the subclavian vein and positioned in the RA appendage and right ventricular apex, respectively, under fluoroscopic guidance. Ten days post-implantation, atrioventricular (AV) node ablation was performed using an 8-mm tip radiofrequency catheter (Blazer II XP, Boston Scientific Corporation, Marlborough, USA) via the femoral vein using an 8-French introducer (ENGAGE Introducer Sheaths, Boston Scientific, Marlborough, USA). Under fluoroscopic guidance, 70W radiofrequency energy was applied to achieve complete AV block, confirmed by intracardiac electrograms and surface electrocardiogram showing AV dissociation (success rate: 92%). Following ablation, high-rate atrial burst pacing (30 s at 20 Hz, twice the diastolic threshold) with intermittent 6-s sensing periods was initiated. If the pacemaker detected sinus rhythm restoration during the sensing period, high-rate atrial pacing was automatically reinitiated to induce a new AF episode. Ventricular pacing was programmed using a rate modulation mode between 60-110 beats per minute. Atrial electrograms during AF episodes were recorded and stored using the automatic switch-mode algorithm of the pacemaker device, which enabled the generation of AF burden curves and the monitoring of the progression from initial self-terminating AF episodes to sustained persistent AF episodes.

Animals in the LAI-AF group were maintained under these conditions for 6 months to ensure the development of sustained persistent AF, whereas animals in the LAI-sr group did not undergo pacemaker implantation or pacing procedures and were maintained over the same follow-up period. Biopsies from the atrium were obtained at the end of follow-up for rhythm-related and NOD1 analyses and were analyzed separately from the main LAI cohort.

Cardiac Magnetic Resonance Imaging

CMR imaging was conducted at the Spanish National Cardiovascular Research Centre (CNIC, Madrid, Spain) using a 3-T Philips Achieva Tx whole-body scanner (Philips Healthcare, Best, Netherlands) equipped with a 32-element phased-array cardiac coil. Scans were performed at 8 weeks (LAI) or 32 weeks (AoB), following established protocols [9]. Standard cine steady-state free-precession (SSFP) sequences were acquired with the following parameters: field of view, 280x280 mm; slice thickness, 6 mm (no gaps); repetition time, 2.8 ms; echo time, 1.4 ms; flip angle, 45°; cardiac phases, 30; voxel size, 1.8 x 1.8 mm; and number of excitations, 3. Forward stroke volume was quantified from 2D flow phase-contrast images of the ascending aorta using a velocity-encoded gradient echo sequence, with the minimum upper velocity limit set to avoid signal aliasing. Parameters for 2D flow imaging included: repetition time/echo time, 5.4/3.4 ms; two signal averages; slice thickness, 8 mm; voxel size, 2.5 × 2.5 mm; and 40 reconstructed cardiac phases. CMR images were independently analyzed by two experienced observers using MR Extended Work Space 2.6 (Philips Healthcare, Best, Netherlands) and QMass MR 7.5 (Medis, Leiden, Netherlands) software. Atrial volumes and function were quantified by measuring atrial area from 4- and 2-chamber views. The following phasic parameters were derived:

- Reservoir strain (%): 100 × (maximal LA area − minimal LA area)/minimal LA area
- Conduit strain (%): 100 × (maximal LA area − pre-atrial contraction area)/maximal LA area

- Contractile strain (%): 100 × (pre-atrial contraction area − minimal LA area)/pre-atrial contraction area

Mouse Studies

All animal procedures conformed to the EU Directive 2010/63/EU and the Spanish regulations for animal experimentation (2013/175) and were approved by the Institutional Animal Care and Use Committee and the General Direction of Agriculture and Environment (PROEX 053/18). All procedures were conducted in accordance with the National Institutes of Health Guide for the Care and Use of Laboratory Animals.

Mouse models

Wild-type (WT; The Jackson Laboratory, Bar Harbor, ME, USA) and *Nod1* knockout (*Nod1*$^{-/-}$; 129P2-*Nod1*^{1tm1Nnz}/J) male mice on a C57BL/6J background were used. *Nod1*$^{-/-}$ mice were generated by replacing exons 1 and 2 of *Nod1* with a neomycin resistance cassette via homologous recombination, as previously described [10], and were kindly provided by G. Núñez et al. (University of Michigan). Heterozygous mice were intercrossed to obtain homozygous *Nod1*$^{-/-}$ mice and WT littermates. Genotyping was performed by PCR using allele-specific primers (Figure S1), and genotypes were independently verified by two blinded observers.

RyR2-S2814A mice (C57BL/6 background) were generated by site-directed mutagenesis of serine 2814 to alanine in exon 56 of the *Ryr2* gene using homologous recombination, as previously described [11]. Heterozygous mice were intercrossed to obtain homozygous RyR2-S2814A mice and WT littermates.

Animals were housed under controlled temperature (23–25°C) and a 12-h light/dark cycle, with ad libitum access to standard chow and water.

Transverse Aortic Constriction (TAC)

Ten-week-old male WT and *Nod1*$^{-/-}$ mice underwent TAC under ketamine/xylazine anesthesia (100/10 mg/kg, i.p.). Anesthesia was confirmed by loss of the withdrawal reflex. Mice underwent trans-sternal thoracotomy, and the transverse aorta was constricted using a 6-0 silk suture tied against a 27-gauge needle, as described previously [12]. Sham-operated mice underwent the same procedure without constriction. Subcutaneous buprenorphine (1 mg/kg) was administered for perioperative analgesia. After 4 weeks, mice were euthanized with intraperitoneal sodium pentobarbital (100 mg/kg) and heparin (4 U/g), followed by LA tissue collection. The atrial tissue was processed for confocal microscopy, proteomic, phosphoproteomic, histological, and molecular analyses.

Pharmacological Treatments

A subset of WT-Sham and WT-TAC mice was treated with the selective NOD1 inhibitor ML-130 (ML; 2 mg/kg, i.p.; Cayman Chemical, Ann Arbor, MI, USA) every other day for 4 weeks. This treatment regimen was selected to achieve sustained NOD1 inhibition throughout the pressure-overload period.

In parallel, healthy 3-month-old male WT*,* *Nod1*$^{-/-}$ and RyR2-S2814A mice were randomized to receive either saline or the NOD1 agonist lauroyl-γ-D-glutamyl-meso-diaminopimelic acid (C12-iE-DAP, 3.3 mg/kg, i.p., Invivogen, San Diego, CA, USA), as previously optimized to achieve robust short-term NOD1 signaling activation [13]. WT mice additionally received the CaMKII inhibitor KN-93 (2.7 mg/kg, i.p., Calbiochem, San Diego, CA, USA), administered either alone or in combination with C12-iE-DAP. All short-term pharmacological treatments were administered once daily for 3 consecutive days, and mice were euthanized on day 4 for atrial tissue collection. Male mice were used to minimize hormonal variability and to ensure consistency with prior studies.

Intracardiac electrophysiology in mice

Atrial and ventricular intracardiac electrograms were recorded using a 1.1F octapolar catheter (EPR-800; Millar Instruments, Houston, TX, USA) inserted via the right jugular vein, as previously described [14]. In brief, right atrial pacing was performed using 2-ms current pulses delivered by an external stimulator (STG-3008; Multi Channel Systems, Reutlingen, Germany). A computer-based data acquisition system (emka TECHNOLOGIES, Paris, France) was used to record a 6-lead body surface ECG and up to 4 intracardiac bipolar electrograms. Surface and intracardiac electrophysiology parameters were assessed at baseline, as described previously [14].

Inducibility of AF was determined using the protocol described by Verheule et al. [15] and was considered positive if at least 2 of 3 pacing trials induced AF. AF was defined as the occurrence of rapid and fragmented atrial electrograms with irregular AV conduction and ventricular rhythm for at least 1 s. A subset of WT mice treated with saline or C12-iE-DAP was used for intracardiac electrophysiological studies (n = 7 per group). These animals were independent from those used for echocardiography and molecular analyses.

<u>CMR and Echocardiography</u>

CMR imaging was conducted on WT and *Nod1*$^{-/-}$ mice (Sham and TAC groups) at the Sols-Morreale Biomedical Research Institute (Madrid, Spain) using a 7.0 T Bruker Pharmascan (Bruker, Ettlingen, Germany). Imaging parameters included an echo time of 1.26 ms, field of view 3 × 3 cm^2, slice thickness 0.8 mm, and a matrix size of 256 × 256, zero-filled to 512 × 512. Mice were anesthetized with 2% isoflurane in oxygen and positioned prone inside a 3.5-cm birdcage radiofrequency coil. Body temperature was maintained at 36 ºC, and heart and respiratory rates were monitored using a SAM system (SA Instruments, Inc., New York, USA). Cardiac- and respiratory-triggered images were acquired, with up to five slices per cardiac cycle, with repetition times adjusted based on physiological parameters. LA and RA volumes were quantified from cine long-axis four-chamber images using manual endocardial contouring

and automatic volumetric calculation with Segment software (Medviso, Lund, Sweden). Atrial endocardial borders were traced at maximal and minimal atrial dimensions, corresponding to ventricular systole and diastole, respectively. The atria were anatomically defined from the AV annular plane to the atrial roof, excluding pulmonary veins and venae cavae and including the atrial appendages. Reservoir function was calculated as 100 x $(V_{max}-V_{min})/V_{max}$. All atrial segmentations were performed blinded to genotype and treatment group.

Echocardiography was performed on WT mice at day 4 post-treatment with saline or C12-iE-DAP using a Vevo 2100 (VisualSonics, Toronto, Canada) equipped with a 30-MHz probe. Mice were lightly anesthetized with 1.5–2% isoflurane and placed on a heated platform to maintain body temperature at 37.0 ± 0.5 °C. Standard B-mode and M-mode images were acquired for LV and LA assessment. Data were analyzed using Vevo LAB software.

Atrial Myocyte Isolation and Ca^{2+} Imaging

Atrial cardiomyocytes were isolated from WT and *Nod1*$^{-/-}$ mice (Sham and TAC groups) and WT-Sham and WT-TAC mice 4 weeks after treatment with the NOD1 inhibitor ML-130. Additionally, atrial cardiomyocytes were obtained from WT, *Nod1*$^{-/-}$ and RyR2-S2814A mice on day 4 following treatment with saline or C12-iE-DAP, as well as from WT mice treated with KN-93 alone or in combination with C12-iE-DAP, as previously described [16–19]. Briefly, hearts were excised after intraperitoneal administration of pentobarbital (100 mg/kg) and heparin (4 U/g). The hearts were immediately immersed in Tyrode's solution (140 mM NaCl, 5.4 mM KCl, 1.0 mM $MgCl_2$, 10 mM glucose, 5 mM HEPES, pH 7.4, 37°C). Atrial tissue was dissected, minced, and enzymatically digested with Liberase TH (0.2 mg/mL, Roche, Mannheim, Germany) in Tyrode's solution supplemented with 0.2 mM $CaCl_2$. Following digestion, tissue fragments were washed in modified Kraft-Brühe solution (100 mM potassium glutamate, 10 mM potassium aspartate, 25 mM KCl, 10 mM KH_2PO_4, 2 mM $MgSO_4$, 20 mM taurine, 5 mM creatine, 0.5 mM EGTA, 20 mM glucose, 5 mM HEPES, 0.1%

BSA, pH 7.2, 37 °C) and mechanically dissociated into single atrial cardiomyocytes. Cells were maintained in Tyrode's solution containing 1 mM Ca^{2+} and used for confocal microscopy within 3 h of isolation.

Isolated atrial cardiomyocytes were loaded with 5 µM Cal-520™ AM (Santa Cruz Biotechnology, Dallas, TX, USA) for 30 min at room temperature. Ca^{2+} transients were recorded in cells paced at 1 Hz via field stimulation using parallel platinum electrodes. Spontaneous Ca^{2+} sparks were imaged in quiescent cardiomyocytes following Ca^{2+} transients acquisition. Sarcoplasmic reticulum (SR) Ca^{2+} load was assessed by rapid application of 10 mM caffeine, after achieving steady state pacing at 1 Hz. Imaging was performed on a Zeiss LSM 710 confocal microscope using a 40× oil immersion objective (1.2 NA) with an argon laser scanning every 1.54 ms. Cal-520™ AM was excited at 493 nm, and emitted fluorescence was collected at >515 nm. Data were processed using IDL software (Research System Inc., Boulder, Colorado, USA), with background fluorescence corrected. Fluorescence values (F) were normalized to baseline fluorescence (F_0) to calculate F/F_0 ratios. SR Ca^{2+} load was quantified as the amplitude of the caffeine-induced Ca^{2+} transient (F/F_0). Ca^{2+} spark properties were analyzed using the SparkMaster plugin in ImageJ (National Institutes of Health, Bethesda, Maryland, USA), with images acquired at 654 lines/s, a pixel size of 0.13 µm, and a detection threshold set at 3.3× the standard deviation (1 interval). Total spark-mediated Ca^{2+} leak was calculated as the product of spark frequency, peak amplitude, duration, and width. Cardiomyocyte surface area was measured using optical microscopy and quantified with the LSM Zeiss Image Browser (Carl Zeiss Microscopy GmbH, Jena, Germany).

Proteomics and Phosphoproteomics

Protein Extraction

LA protein lysates from WT and *Nod1*$^{-/-}$ mice (Sham and TAC groups) were prepared from frozen atrial tissue homogenized with ceramic beads (MagNa Lyser Green Beads, Roche,

Mannheim, Germany) in extraction buffer (50 mM Tris-HCl, 1 mM EDTA, 1.5% SDS, 50 mM iodoacetamide, pH 8.5). Protein concentrations were quantified using the RC DC Protein Assay (Bio-Rad, Hercules, CA, USA). Samples underwent tryptic digestion using a filter-assisted sample preparation (FASP) protocol (Expedeon, San Diego, CA, USA), as described previously [20]. Proteins were digested overnight at 37 °C with sequencing-grade trypsin (Promega, Madison, WI, USA) at a 1:40 enzyme-to-protein ratio in 50 mM ammonium bicarbonate (pH 8.5). Peptides were recovered by centrifugation, acidified with 1% trifluoroacetic acid (TFA, v/v), desalted on C18 Oasis HLB cartridges (Waters Corporation, Milford, MA, USA), and dried under vacuum.

Isobaric Peptide Labeling

Peptides were labeled using TMTpro 18plex reagents (Thermo Fisher Scientific, San Jose, CA, USA). Each 18-plex batch included 17 biological samples and one pooled internal reference (WT-Sham). Peptides were dissolved in 1 M triethylammonium bicarbonate, and concentrations determined by infrared spectroscopy (Direct Detect, Millipore, Billerica, MA, USA). Equal peptide amounts were labeled according to the manufacturer's protocol, combined, and vacuum-dried prior to fractionation.

High-pH Reversed-Phase Fractionation

TMT-labeled peptides were reconstituted in 0.1% TFA and fractionated into five fractions using the high pH reversed-phase peptide fractionation kit (Thermo Scientific, Waltham, MA, USA). Spin columns were equilibrated with acetonitrile, followed by two washes with 0.1% trifluoroacetic acid. Samples were loaded, centrifuged at 3,000 × g for 2 min, and washed. Peptides were eluted with: (1) 12.5% acetonitrile, 87.5% triethylamine; (2) 15% acetonitrile, 85% triethylamine; (3) 17.5% acetonitrile, 82.5% triethylamine; (4) 20% acetonitrile, 80% triethylamine; and (5) 50% acetonitrile, 50% triethylamine. Fractions were dried and stored at −20°C until mass spectrometry analysis.

Phosphopeptide Enrichment

Phosphopeptides were enriched using Ti-IMAC magnetic microparticles (MagReSyn, Resyn Biosciences, Edenvale, South Africa) [21]. Labeled peptides were resuspended in loading buffer (80% acetonitrile, 5% TFA, 1 M glycolic acid) and incubated with Ti-IMAC beads for 20 min. Beads were sequentially washed with: (i) loading buffer; (ii) 80% acetonitrile, 1% TFA; and (iii) 10% acetonitrile, 0.2% TFA. Phosphopeptides were eluted with 1% ammonia for 10 min and dried for LC–MS/MS. Unbound fractions underwent a second enrichment cycle.

Liquid Chromatography-Tandem Mass Spectrometry (LC–MS/MS)

Samples were analyzed using an Easy-nanoLC 1000 (Thermo Fisher Scientific, Waltham, MA, USA) coupled to an Orbitrap Fusion Trihybrid Mass Spectrometer (Thermo Fisher Scientific, Waltham, MA, USA). Peptides were loaded onto an Acclaim PepMap 100 C18 trap column (2 cm × 75 µm) and separated on a PepMap RSLC C18 EASY-Spray analytical column (50 cm × 75 µm). Peptides were eluted with a 300-min linear gradient from 0.1% formic acid in water to 100% acetonitrile with 0.1% formic acid at 200 nL/min. Full MS scans were acquired in a data-dependent manner, switching between MS and MS/MS with a 3 s TopSpeed method with 40 s dynamic exclusion. MS spectra were collected in full ion-scan mode (400-1500 m/z) at 120,000 resolution, with an automatic gain control target of 2×10^5 and 50 ms maximum injection time. Higher-energy collisional dissociation was performed at 35% of normalized collision energy, and MS/MS spectra were analyzed at 30,000 resolution, an AGC target of 5×10^5 and, 120 ms maximum injection time.

Peptide and Protein Identification

Raw data were processed using SEQUEST HT (Proteome Discoverer 2.5, Thermo Fisher Scientific, Waltham, MA, USA) [22] against the *Mus musculus* UniProtKB database (June 2023) [23] concatenated with decoy sequences (DecoyPyrat) [24]. Trypsin digestion was specified with up to two missed cleavages. Dynamic modifications included cysteine

carbamidomethylation (+57.021 Da), cysteine methylthiolation (+45.988 Da), serine/threonine/tyrosine phosphorylation (+79.966 Da), and methionine oxidation (+15.995 Da). Fixed modifications included TMT labeling at the N-terminus and lysine (+304.207 Da). Precursor and fragment mass tolerance were set at 8 ppm and 0.02 Da, respectively, with a precursor charge range to 2-4. False discovery rate (FDR) was calculated using the corrected Xcorr score (cXcorr) [25] and a target/decoy strategy with the picked FDR method at the peptide level [26], refined by a 15 ppm precursor mass tolerance filter [27]. A 1% FDR threshold was applied for peptide identification.

Peptides were assigned to proteins using an in-house algorithm. Proteins were ranked by the number of identified peptides, and each peptide was assigned to protein with the most peptides. Ties were resolved by prioritizing proteins with more peptide-spectrum matches.

Quantification and Functional Analysis

TMT reporter intensities were integrated from spectra to peptides and protein using the Weighted Spectrum, Peptide, Protein (WSPP) model [28] and Generic Integration Algorithm (GIA)[29] via iSanXoT software [30,31]. Protein abundance was expressed as standardized Zq values (normalized $\log_2$ ratios in standard deviation units). Differential protein abundance was assessed using the Limma package, with p-values adjusted for multiple testing via FDR. Proteins identified with ≥ 2 peptides, $|Zq| > 1$, and FDR < 0.05 were considered statistically significantly altered. Peptide-level quantifications were derived from Zpq values (protein-corrected $\log_2$ ratios; Tukey-adjusted $p < 0.05$) [32]. Functional categories were analyzed using the Systems Biology Triangle algorithm [29], calculating standardized $\log_2$ fold-changes (*Zc* values). Proteins were annotated using the DAVID repository [33], including Gene Ontology and PANTHER databases. Functional category changes were deemed significant at FDR < 5%.

Western Blotting

Atrial tissue from humans, pigs, and mice was homogenized in RIPA buffer (25 mM Tris-HCl, pH 7.6, 150 mM NaCl, 1% NP-40, 1% sodium deoxycholate, 0.1% SDS; Thermo Fisher Scientific, Waltham, MA, USA) supplemented with protease/phosphatase inhibitors and okadaic acid (Sigma-Aldrich, St. Louis, MO, USA). Homogenates were centrifuged (13,000 rpm, 15 min, 4 °C), and supernatant protein concentrations were determined using the bicinchoninic acid assay (Thermo Fisher Scientific, Waltham, MA, USA). Samples were prepared for SDS-PAGE by adding loading buffer containing 10% β-mercaptoethanol and heated at 90 °C for 5 min (or 60 °C for 15 min for Ca^{2+}-handling proteins). Proteins were separated on SDS–PAGE gels (BioRad, Hercules, CA, USA) and transferred to PVDF membranes using a semi-dry transfer system. Membranes were blocked in TBS-T (50 mM Tris-HCl, 150 mM NaCl, 0.1% Tween-20) containing 5% BSA and incubated overnight with the primary antibodies (Table S5). Following incubation with HRP-linked secondary antibodies (1:5,000) in TBS-Tween-20 buffer for 1 h at room temperature, immunoreactive bands were visualized using SuperSignal™ West Femto Maximum Sensitivity Substrate (Thermo Fisher Scientific, Waltham, MA, USA). Band intensity was quantified using ImageJ software (National Institutes of Health, Bethesda, Maryland, USA). Protein levels were normalized to total protein loading or housekeeping proteins as indicated in the corresponding figure legends.

Total RNA Isolation and Quantitative Real-Time PCR

Total RNA was extracted from atrial samples of pigs and mice using Qiazol and a TissueLyser LT, with purification on MinElute columns (Qiagen, Germantown, MD, USA). RNA integrity was evaluated using an RNA Nano Chip (Agilent Technologies, Santa Clara, CA, USA), and concentrations were measured with a NanoDrop spectrophotometer (NanoDrop Technologies, Wilmington, DE, USA). cDNA was synthesized using the High-Capacity cDNA Reverse Transcription Kit (Thermo Fisher Scientific, Waltham, MA, USA). qPCR was performed on a

7900HT Fast Real-Time PCR System (Applied Biosystems, Foster City, CA, USA). Relative mRNA levels were normalized to *Rplp0* (36B4, mouse) or *GAPDH* (pig) using the $2^{(\Delta\Delta Ct)}$ method. Primer sequences are listed in Table S6. PCR efficiency and specificity were confirmed by melt-curve analysis.

Histological Analysis

Hematoxylin–Eosin (H&E) and Masson's trichrome staining

Atrial myocardium samples from patients, mice and pigs were fixed in 4% neutral buffered formalin for 12 h at room temperature, dehydrated, embedded in paraffin, and sectioned into 5-µm slices. Routine hematoxylin-eosin (H&E) staining was performed on human, swine, and mouse atrial tissue while Masson's trichrome staining was performed on human and murine atrial samples. Images were captured with an Olympus BX41 microscope (Olympus Corporation, Center Valley, PA, USA) and processed with ImagePro Plus 5.3 (Media Cybernetics, Rockville, MD, USA).

Immunohistochemistry

Five-micrometer sections of atrial myocardium from patients, pigs, and mice were subjected to NOD1 immunostaining. Immunohistochemistry for inflammatory cell markers (CD3, CD19, and CD68) was performed on atrial tissue from all experimental groups. Antigen retrieval was conducted using pH 6.0 citrate buffer in a PT-Link system (Dako, Carpinteria, CA, USA) followed by quenching of endogenous peroxidase and blocking (10% normal goat serum, 1% BSA, 0.01% Triton X-100). Sections were incubated overnight at 4 °C with primary antibodies: NOD1 (1:200, GeneTex, Irvine, CA, USA), CD68 (1:200, Novus Biologicals, Centennial, CO, USA), CD3 (1:500, Thermo Fisher Scientific, Waltham, MA, USA), CD19 (1:1000, Thermo Fisher Scientific, Waltham, MA, USA). After secondary antibody incubation (1 h, room temperature; Sigma-Aldrich, St. Louis, MO, USA), signal was visualized using a DAB substrate (Palex, Barcelona, Spain), and sections were counterstained with Mayer's

hematoxylin (Merck Millipore, Burlington, MA, USA). Negative controls employed isotype antibody (Thermo Fisher Scientific, Waltham, MA, USA). Images were captured with an Olympus BX41 microscope (Olympus Corporation, Center Valley, PA, USA) and processed with ImagePro Plus 5.3 (Media Cybernetics, Rockville, MD, USA). Immune cell infiltration and NOD1 staining were evaluated by examining at least five randomly selected fields per section using ImageJ software (National Institutes of Health, Bethesda, Maryland, USA).

References

1. Nagueh SF, et al. Recommendations for the evaluation of left ventricular diastolic function by echocardiography: an update from the American Society of Echocardiography and the European Association of Cardiovascular Imaging. Eur Heart J Cardiovasc Imaging. 2016; 17: 1321–60.

2. Badano LP, et al. Standardization of left atrial, right ventricular, and right atrial deformation imaging using two-dimensional speckle tracking echocardiography: a consensus document of the EACVI/ASE/Industry Task Force to standardize deformation imaging. Eur Heart J Cardiovasc Imaging. 2018; 19: 591–600.

3. Jiang H, et al. Skewer: a fast and accurate adapter trimmer for next-generation sequencing paired-end reads. BMC Bioinformatics. 2014; 15: 182.

4. Dobin A, Gingeras TR. Mapping RNA-seq reads with STAR. Curr Protoc Bioinformatics. 2015; 51: 11.14.1–11.14.19.

5. Li B, Dewey CN. RSEM: accurate transcript quantification from RNA-Seq data with or without a reference genome. BMC Bioinformatics. 2011; 12: 323.

6. Pertea M, et al. StringTie enables improved reconstruction of a transcriptome from RNA-seq reads. Nat Biotechnol. 2015; 33: 290–5.

7. Wang L, et al. DEGseq: an R package for identifying differentially expressed genes from RNA-seq data. Bioinformatics. 2010; 26: 136–8.

8. Ayaon-Albarran A, et al. Systolic flow displacement using 3D magnetic resonance imaging in an experimental model of ascending aorta aneurysm: impact of rheological factors. Eur J Cardiothorac Surg. 2016; 50: 685–92.

9. Fernández-Jiménez R, et al. Myocardial Edema After Ischemia/Reperfusion Is Not Stable and Follows a Bimodal Pattern: Imaging and Histological Tissue Characterization. J Am Coll Cardiol. 2015; 65: 315–23.

10. Chamaillard M, et al. An essential role for NOD1 in host recognition of bacterial peptidoglycan containing diaminopimelic acid. Nat Immunol. 2003; 4: 702–7.

11. Chelu MG, et al. Calmodulin kinase II-mediated sarcoplasmic reticulum Ca^{2+} leak promotes atrial fibrillation in mice. J Clin Invest. 2009; 119: 1940–51.

12. Rockman HA, et al. Segregation of atrial-specific and inducible expression of an atrial natriuretic factor transgene in an *in vivo* murine model of cardiac hypertrophy. Proc Natl Acad Sci U S A. 1991; 88: 8277–81.

13. Delgado C, et al. NOD1, a new player in cardiac function and calcium handling. Cardiovasc Res. 2015; 106: 375–86.

14. Sood S, et al. Intracellular calcium leak due to FKBP12.6 deficiency in mice facilitates the inducibility of atrial fibrillation. Heart Rhythm. 2008; 5: 1047–54.

15. Verheule S, et al. Increased vulnerability to atrial fibrillation in transgenic mice with selective atrial fibrosis caused by overexpression of TGF-beta1. Circ Res. 2004; 94: 1458–65.

16. Jansen HJ, et al. Regional and temporal progression of atrial remodeling in angiotensin II mediated atrial fibrillation. Front Physiol. 2022; 13: 1021807.

17. Jansen HJ, Rose RA. Isolation of atrial myocytes from adult mice. J Vis Exp. 2019; 149: e59588.

18. Jansen HJ, et al. Distinct patterns of atrial electrical and structural remodeling in angiotensin II mediated atrial fibrillation. J Mol Cell Cardiol. 2018; 124: 12–25.

19. Egom EE, et al. Impaired sinoatrial node function and increased susceptibility to atrial fibrillation in mice lacking natriuretic peptide receptor C. J Physiol. 2015; 593: 1127–46.

20. Bonzon-Kulichenko E, et al. Improved integrative analysis of the thiol redox proteome using filter-aided sample preparation. J Proteomics. 2020; 214: 103624.

21. Bekker-Jensen DB, et al. Rapid and site-specific deep phosphoproteome profiling by data-independent acquisition without the need for spectral libraries. Nat Commun. 2020; 11: 787.

22. Orsburn BC. Proteome discoverer—a community enhanced data processing suite for protein informatics. Proteomes. 2021; 9: 15.

23. Bateman A, et al. UniProt: the universal protein knowledgebase in 2023. Nucleic Acids Res. 2023; 51: D523–31.

24. Wright JC, Choudhary JS. DecoyPyrat: fast non-redundant hybrid decoy sequence generation for large scale proteomics. J Proteomics Bioinform. 2016; 9: 176–80.

25. Keller A, et al. Empirical statistical model to estimate the accuracy of peptide identifications made by MS/MS and database search. Anal Chem. 2002; 74: 5383–92.

26. Prieto G, Vázquez J. Protein probability model for high-throughput protein identification by mass spectrometry-based proteomics. J Proteome Res. 2020; 19: 1285–97.

27. Bonzon-Kulichenko E, et al. Revisiting peptide identification by high-accuracy mass spectrometry: problems associated with the use of narrow mass precursor windows. J Proteome Res. 2015; 14: 700–10.

28. Navarro P, et al. General statistical framework for quantitative proteomics by stable isotope labeling. J Proteome Res. 2014; 13: 1234–47.

29. García-Marqués F, et al. A novel systems-biology algorithm for the analysis of coordinated protein responses using quantitative proteomics. Mol Cell Proteomics. 2016; 15: 1740–60.

30. Trevisan-Herraz M, et al. SanXoT: a modular and versatile package for the quantitative analysis of high-throughput proteomics experiments. Bioinformatics. 2019; 35: 1594–6.

31. Rodríguez JM, et al. iSanXoT: a standalone application for the integrative analysis of mass spectrometry-based quantitative proteomics data. Comput Struct Biotechnol J. 2023; 23: 452–9.

32. Bagwan N, et al. Comprehensive quantification of the modified proteome reveals oxidative heart damage in mitochondrial heteroplasmy. Cell Rep. 2018; 23: 3685–97.e4.

33. Huang DW, Sherman BT, Lempicki RA. Systematic and integrative analysis of large gene lists using DAVID bioinformatics resources. Nat Protoc. 2009; 4: 44–57.

Supplementary Tables

Table S1. Changes in quantitative abundance of RyR2 phosphorylated peptides on atrial tissue from *WT*-Sham and *WT*-TAC mice.

Phosphorylation abundance was determined by the standardized variable (Zpq), expressing the deviation between the log_2-ratio quantifications of each peptide from the protein of origin, using the WSPP statistical model. Phosphorylated Ser, Thr and Tyr sites are indicated in the peptide sequence column. WT-Sham (n=4) and WT-TAC (n=4) mice.

Uniprot protein ID	Gene ID	Peptide	No. of PSMs	WT-Sham				WT-TAC			
E9Q401	*Ryr2*	AALSLPANVEDVCPNLPSLEK;S18(Phospho)	4	-0.2	-0.7	-3.9		-1.1	-2.9	-2.9	-1.6
E9Q401	*Ryr2*	YFTLCGLQEGYEPFAVNTNR;Y11(Phospho)	1			0.07	-0.3		0.6	1.7	
E9Q401	*Ryr2*	LAEDPSRDGPSPTSGSSK;S14(Phospho)	1	-0.7	-0.7			-0.4			1.18
E9Q401	*Ryr2*	RLSQTSQVSLDAAHGYSPR;S3(Phospho)	2	0.85	0.88	-0.1	-0.4	1.91	0.14	1.02	1.83
E9Q401	*Ryr2*	AALSLPANVEDVCPNLPSLEK;S4(Phospho)	2			-1.2	-0.2		-0.7	-1.1	
E9Q401	*Ryr2*	YSMQTSLLVAALK;S2(Phospho)	1	1.44	-0.1			0.57			-0.6

Ryr2, ryanodine receptor 2 gene.

Table S2. Changes in quantitative abundance of RyR2 phosphorylated peptides on atrial tissue from *Nod1*$^{-/-}$-Sham and *Nod1*$^{-/-}$-TAC mice.

Phosphorylation abundance was determined by the standardized variable (Zpq), expressing the deviation between the log_2-ratio quantifications of each peptide from the protein of origin, using the WSPP statistical model. Phosphorylated Ser, Thr and Tyr sites are indicated in the peptide sequence column. *Nod1*$^{-/-}$-Sham (n=4) and *Nod1*$^{-/-}$-TAC (n=5) mice.

Uniprot protein ID	Gene ID	Peptide	No. of PSMs	Nod1$^{-/-}$-Sham				Nod1$^{-/-}$-TAC				
E9Q401	*Ryr2*	AALSLPANVEDVCPNLPSLEK;S18(Phospho)	4	0.11	0.86	-2.7	-0.8	0.43	-1.4	-2.7	-3	-3.2
E9Q401	*Ryr2*	YFTLCGLQEGYEPFAVNTNR;Y11(Phospho)	1			-1.6	-0.5			0.71	-0.3	0.77
E9Q401	*Ryr2*	LAEDPSRDGPSPTSGSSK;S14(Phospho)	1	-0.5	-0.8			-1.7	-0.8			
E9Q401	*Ryr2*	RLSQTSQVSLDAAHGYSPR;S3(Phospho)	2	0.23	-0	-1.7	-1.2	0.35	0.76	0.04	0.46	-2.3
E9Q401	*Ryr2*	AALSLPANVEDVCPNLPSLEK;S4(Phospho)	2			-0.8	-0.2			-1.4	-0.9	-1
E9Q401	*Ryr2*	YSMQTSLLVAALK;S2(Phospho)	1	0.86	0.25			0.94	0.43			

Ryr2, ryanodine receptor 2 gene.

Table S3. Macroscopic parameters in ML-130–treated mice subjected to sham surgery or TAC.

Data are mean ± SEM; statistical analyses performed by ANOVA with Tukey's post hoc across all four groups (WT-Sham, WT-TAC, WT-Sham ML, WT-TAC ML); P < 0.05 considered significant; ## P < 0.01, ### P < 0.001 vs. WT-TAC.

	**WT-Sham ML n=3**	**WT-TAC ML n=3**
**Body parameters**		
BW (g)	29.11 ± 1.69	27.97 ± 0.20
TL (mm)	16.97 ± 0.04	16.98 ± 0.03
HW (mg)	195.8 ± 9.22###	169.3 ± 7.94###
LAW (mg)	8.30 ± 1.11###	5.1 ± 0.61###
RAW (mg)	5.27 ± 0.27##	5.23 ± 0.61##
HW/BW (mg/g)	6.75 ± 0.32###	6.04 ± 0.26###
LAW/BW (mg/g)	0.29 ± 0.04###	0.18 ± 0.02###
RAW/BW (mg/g)	0.18 ± 0.002##	0.19 ± 0.02#
HW/TL (mg/mm)	11.54 ± 0.53###	9.95 ± 0.45###
LAW/TL (mg/mm)	0.49 ± 0.07###	0.30 ± 0.03###
RAW/TL (mg/mm)	0.31 ± 0.02##	0.31 ± 0.01##
LA cell area (μm^2)	996.0 ± 33.85###	1175 ± 48.43###

BW, body weight; HW, heart weight; LAW, left atrial weight; RAW, right atrial weight; TL, tibia length.

Table S4. Relative quantification of major gene expression changes in atrial tissue of NF and HF patients.

DESeq2 was applied to transform gene count data (normalized counts) to the $\log_2$ scale, reducing sample variation in low-count rows and normalizing by library size. NF (n=3) and HF (n=3). lfcSE, standard error estimate for the log2 fold change estimate.

Gene ID	Gene	log2FoldChange	lfcSE	NF			HF		
ENSG00000034971	*MYOC*	-2.00	0.58	8.08	9.04	8.39	7.04	7.05	7.77
ENSG00000152580	*IGSF10*	-1.41	0.40	8.97	8.61	8.96	7.84	8.17	7.93
ENSG00000137441	*FGFBP2*	-1.72	0.50	6.81	7.01	7.49	6.08	6.15	6.30
ENSG00000215018	*COL28A1*	-2.45	0.63	5.61	5.60	6.02	4.57	4.50	4.88
ENSG00000197576	*HOXA4*	-2.81	0.80	4.53	4.57	5.03	3.82	3.63	3.92
ENSG00000105697	*HAMP*	-2.21	0.60	11.63	11.60	12.26	10.98	9.75	10.23
ENSG00000125851	*PCSK2*	-2.52	0.52	6.31	6.83	6.71	5.53	5.22	5.28
ENSG00000164920	*OSR2*	-1.91	0.53	5.91	6.24	6.28	5.35	5.05	5.24
ENSG00000158887	*MPZ*	-2.22	0.59	6.64	7.59	7.59	6.39	5.97	5.85
ENSG00000281887	*GIMAP1-GIMAP5*	-7.81	1.48	3.80	4.44	4.75	3.01	2.94	2.92
ENSG00000118231	*CRYGD*	-7.05	1.59	3.11	3.61	3.93	2.63	2.58	2.57
ENSG00000006327	*TNFRSF12A*	3.43	0.74	6.76	6.71	7.21	9.10	7.35	9.67
ENSG00000169715	*MT1E*	2.10	0.59	9.91	10.15	10.32	11.44	10.45	12.04
ENSG00000125144	*MT1G*	7.28	1.78	3.94	3.85	4.29	6.29	4.35	10.52
ENSG00000099625	*CBARP*	3.37	0.90	4.66	4.33	4.78	5.83	4.83	7.20
ENSG00000148339	*SLC25A25*	2.75	0.74	5.54	5.89	6.11	6.57	6.78	8.24
ENSG00000137309	*HMGA1*	1.78	0.51	8.24	8.49	8.62	9.15	9.26	10.09
ENSG00000034152	*MAP2K3*	1.97	0.57	8.30	8.09	8.36	8.99	8.94	10.13
ENSG00000125148	*MT2A*	2.89	0.67	11.53	11.53	11.51	12.70	12.29	14.24
ENSG00000171051	*FPR1*	2.97	0.80	4.33	4.46	4.44	5.29	4.95	6.49
ENSG00000106366	*SERPINE1*	3.16	0.85	5.22	6.46	5.78	7.22	6.76	8.64
ENSG00000167470	*MIDN*	1.42	0.38	8.13	8.43	8.25	9.03	9.09	9.33
ENSG00000158050	*DUSP2*	3.33	0.94	5.36	5.60	4.92	5.25	6.60	8.22
ENSG00000187479	*C11orf96*	2.60	0.77	9.08	9.47	8.82	9.21	10.37	11.70
ENSG00000109321	*AREG*	4.92	1.19	4.51	4.54	3.83	4.06	6.41	8.19
ENSG00000137801	*THBS1*	4.21	1.06	6.46	6.50	5.62	5.94	7.77	10.11
ENSG00000205362	*MT1A*	3.66	0.97	5.54	5.11	5.32	5.19	6.71	8.52
ENSG00000130844	*ZNF331*	2.89	0.82	7.01	7.03	6.97	6.76	8.50	9.66
ENSG00000136244	*IL6*	10.97	2.44	4.55	4.82	4.45	4.83	8.56	13.79
ENSG00000158859	*ADAMTS4*	7.43	2.09	3.73	4.15	3.52	4.17	6.21	10.13
ENSG00000108700	*CCL8*	5.90	1.64	2.57	2.51	2.36	2.45	3.25	4.89
ENSG00000188042	*ARL4C*	3.18	0.90	5.27	5.24	4.88	5.37	6.04	7.89
ENSG00000115602	*IL1RL1*	4.96	1.33	3.02	3.55	2.90	3.56	4.16	6.15
ENSG00000124762	*CDKN1A*	4.45	0.87	8.39	8.69	8.65	9.39	10.32	12.55
ENSG00000185022	*MAFF*	4.20	0.91	6.48	7.23	6.71	7.37	8.51	10.56
ENSG00000137193	*PIM1*	3.40	0.93	6.65	7.01	6.59	6.94	7.45	10.03
ENSG00000120694	*HSPH1*	3.38	0.97	7.47	7.71	7.29	7.38	8.07	10.85
ENSG00000163874	*ZC3H12A*	4.13	0.97	6.26	6.46	6.87	7.02	7.59	10.34
ENSG00000076604	*TRAF4*	2.83	0.80	5.64	5.41	5.37	5.98	6.22	7.95
ENSG00000123358	*NR4A1*	3.36	0.91	9.52	10.88	8.98	10.65	12.44	12.94
ENSG00000166016	*ABTB2*	1.90	0.55	5.73	6.09	5.77	6.36	7.05	7.15

ENSG00000136997	*MYC*	4.31	1.09	6.93	7.25	5.88	6.22	9.31	10.59
ENSG00000275993	*SIK1B*	3.93	1.03	6.83	7.27	5.68	6.62	9.64	9.84
ENSG00000179388	*EGR3*	3.74	0.98	4.48	5.17	4.21	4.85	6.70	7.40
ENSG00000144655	*CSRNP1*	3.48	0.83	7.94	8.06	6.75	8.58	9.69	10.70
ENSG00000173334	*TRIB1*	2.71	0.79	7.97	7.74	6.81	8.17	8.74	10.17
ENSG00000184557	*SOCS3*	4.03	1.07	6.90	8.41	6.62	7.90	9.35	11.36
ENSG00000103257	*SLC7A5*	2.82	0.72	6.08	6.34	5.64	6.73	7.29	8.43
ENSG00000125538	*IL1B*	5.68	1.16	3.83	3.78	3.35	4.41	5.39	7.41
ENSG00000075426	*FOSL2*	2.70	0.72	8.10	7.95	7.47	8.31	9.11	10.38
ENSG00000119508	*NR4A3*	6.68	1.03	4.33	4.69	4.24	5.40	7.37	9.12
ENSG00000168906	*MAT2A*	2.22	0.65	9.07	9.21	8.66	9.23	10.26	11.10
ENSG00000144596	*GRIP2*	3.15	0.73	5.76	5.60	5.80	8.33	6.97	6.45
ENSG00000225972	*MTND1P23*	7.67	1.89	10.77	11.88	10.95	18.61	16.20	10.65
ENSG00000237973	*MTCO1P12*	6.03	1.72	13.44	13.59	12.41	19.41	15.47	13.00
ENSG00000278558	*TMEM191B*	7.70	1.79	2.25	2.27	2.24	4.45	2.42	3.21
ENSG00000144597	*EAF1*	2.87	0.81	5.83	5.52	5.05	7.89	5.88	6.78
ENSG00000224420	*ADM5*	3.56	1.04	3.04	3.33	3.26	4.43	3.78	4.19
ENSG00000206561	*COLQ*	3.32	0.72	5.84	6.21	6.10	8.86	7.09	7.23
ENSG00000175832	*ETV4*	3.00	0.81	4.19	4.45	4.80	6.52	5.15	5.66
ENSG00000120937	*NPPB*	5.13	0.87	11.71	10.48	11.20	15.50	12.92	13.83
ENSG00000115457	*IGFBP2*	2.90	0.62	9.38	8.88	9.20	11.77	10.29	10.40
ENSG00000181773	*GPR3*	3.61	0.84	3.90	4.01	4.53	6.13	5.35	5.57
ENSG00000184545	*DUSP8*	2.24	0.53	6.80	6.80	7.24	8.79	8.01	7.94
ENSG00000131389	*SLC6A6*	2.73	0.73	7.45	6.34	5.99	8.32	8.63	8.40
ENSG00000198113	*TOR4A*	1.97	0.50	5.91	5.81	5.76	7.04	7.08	6.47
ENSG00000078804	*TP53INP2*	1.19	0.35	9.72	9.62	9.58	10.46	10.49	10.22
ENSG00000163217	*BMP10*	3.08	0.79	6.97	6.58	6.19	7.13	9.40	7.89
ENSG00000015413	*DPEP1*	7.25	1.89	1.94	1.96	1.94	2.40	3.61	2.07
ENSG00000127533	*F2RL3*	3.23	0.94	3.90	4.03	3.96	4.78	6.18	4.21

Table S5. **Antibodies and their descriptions**

Antibody	Concentration	Host	Reference	Company
**NOD1**	1:500	Rabbit	3545S	Cell Signaling
**RIP2**	1:2000	Rabbit	GTX31647	GeneTex
**IL1β**	1:500	Rabbit	NB600-633	Novus Biologicals
**IL6**	1:1000	Rabbit	P260	Thermo Fisher Scientific
**pRyR2 (Ser2814)**	1:2000	Rabbit	A010-31	Badrilla
**RyR2**	1:2500	Mouse	MA3-916	Thermo Fisher Scientific
**pCaMKII (Thr286/287)**	1:1000	Rabbit	3361	Cell Signaling
**CaMKII**	1:1000	Rabbit	3362	Cell Signaling
**SERCA2a**	1:500	Mouse	sc-53010	Santa Cruz Biotechnology
**GAPDH**	1:10000	Mouse	AM4300	Thermo Fisher Scientific

Table S6. **Oligonucleotide sequences and their descriptions**

Gene	Forward primer	Reverse primer
**pig-*IL6***	GGATTTCCTGCAGTTCAGCCT	CAGGTTTCTGACCAGAGGAGG
**pig-*IL1B***	AATTCAGGGACCCTACCCTCTC	CCATCACTTCCTTGGCGGG
**pig-*GAPDH***	TGCACCACCAACTGCTTAGC	GGCATGGACTGTGGTCATGAG
**mouse-*Nod1***	CCTTGCTTTAGCCGTCTCAC	TCCTCACATAGCACCTTCACC
**mouse-*Rip2***	ATGCCACCTGAGAACTATGAGC	GCAAAGGATTGGTGACCTCTT
**mouse-*Il6***	GAGGATACCACTCCCAACAGACC	AAGTGCATCATCGTTGTTCATACA
**mouse-*Il1b***	CACCTCTCAAGCAGAGCACAG	GGGTTCCATGGTGAAGTCAAC
**mouse-*Tgfb1***	AGAAGTCACCCGCGTGCTAAT	CACTGCTTCCCGAATGTCTGA
**mouse-*Rplp0* (36B4)**	ACTGGTCTAGGACCCGAGAAG	TCCCACCTTGTCTCCAGTCT

Supplementary Figures

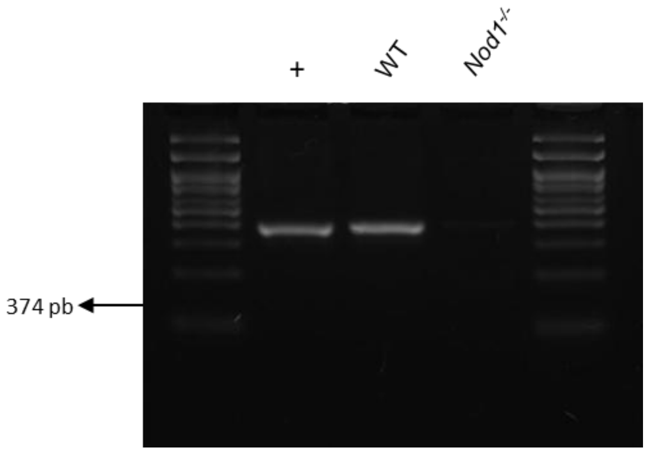

Figure S1. Genotyping in WT and *Nod1*$^{-/-}$ animals. (A) Representative PCR-based genotyping confirming the presence of the *Nod1* allele in WT mice and its deletion in *Nod1*$^{-/-}$ mice, as determined by agarose gel electrophoresis.

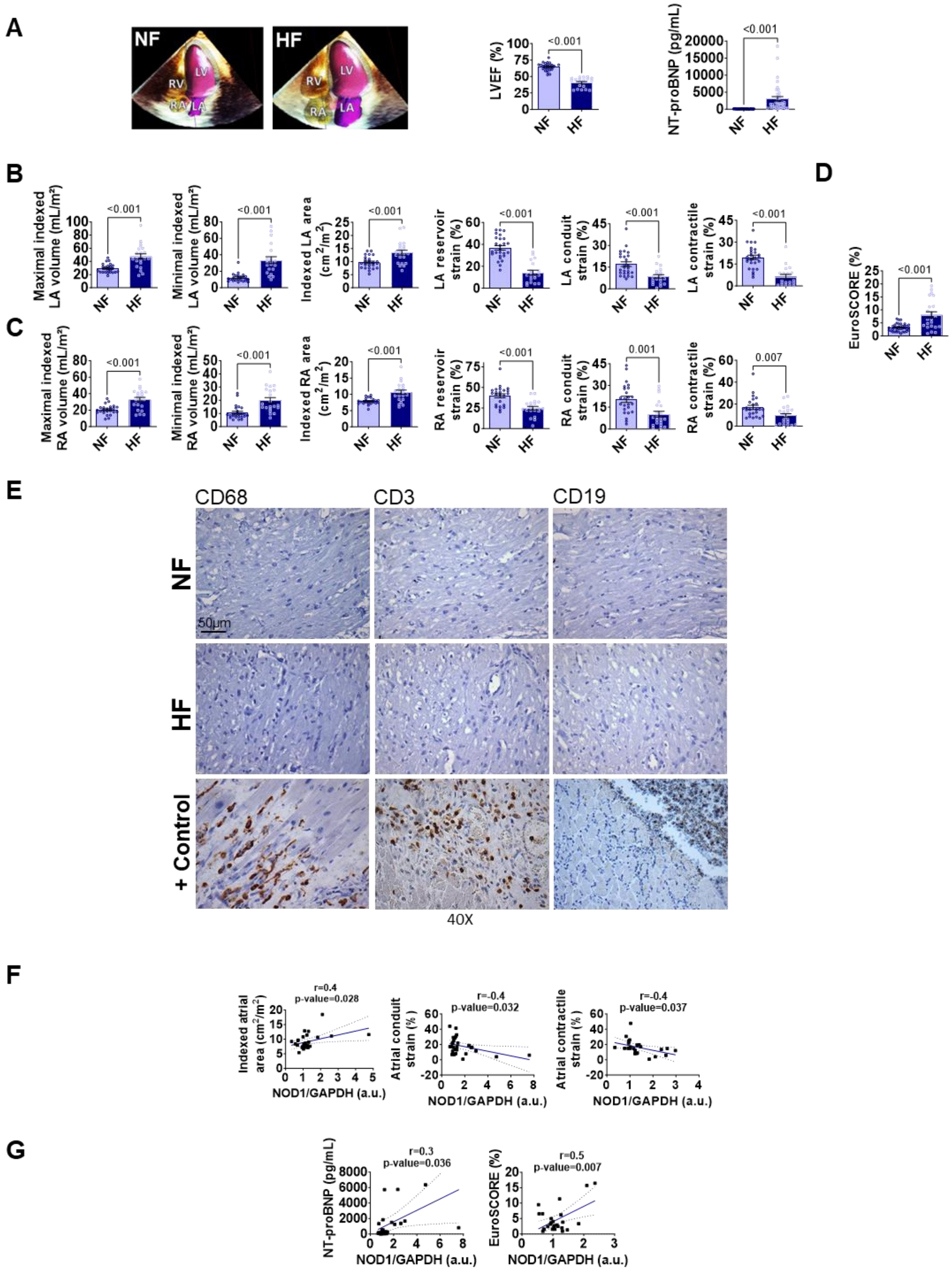

Figure S2. Cardiac and immunohistological features of NF and HF patients: correlation between atrial NOD1 expression and clinical parameters. (A) Representative 3D echocardiographic images from both groups. Left ventricular ejection fraction (LVEF) and NT-

ProBNP levels in non-failing (NF, *n* = 45) and heart failure (HF, *n* = 36) patients. (B-C) Maximal and minimal LA (B) and RA (C) volumes, areas, conduit and contractile strain. (D) Predicted in-hospital mortality risk after surgery (EuroSCORE) in NF (*n* = 26-45) and HF (*n* = 20-36) patients. (E) Representative IHC staining for CD68 (macrophages), CD3 (T lymphocytes), and CD19 (B lymphocytes) in atrial tissue from NF and HF individuals. Representative histological sections of human myocardial tissue were also immunostained for the same markers and served as positive controls to confirm the specificity of the primary antibodies (lower panel). (F-G) Correlations between atrial NOD1 protein levels and indexed atrial area, conduit and contractile strains (F), and NT-proBNP levels and EuroSCORE (G). Data are mean ± SEM; statistical significance determined by unpaired t-test or Pearson's correlation ($P < 0.05$). EuroSCORE, European System for Cardiac Operative Risk Evaluation.

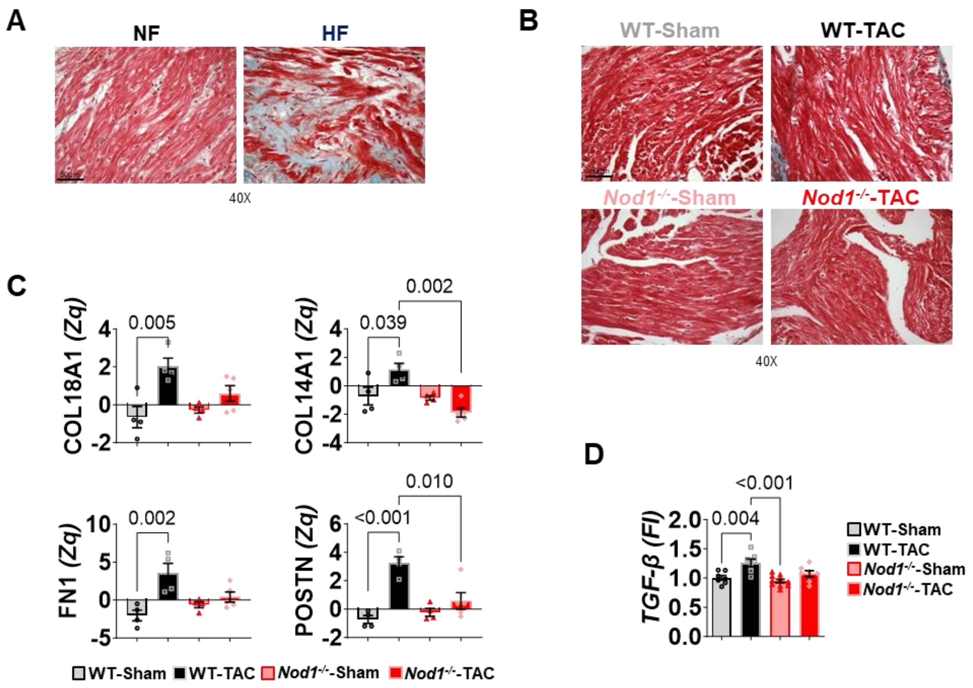

Figure S3. Analysis of extracellular matrix remodeling in human and murine atrial tissue. (A) Masson's Trichrome staining of atrial tissue from NF and HF patients. (B) Masson's Trichrome staining of LA tissue from WT-Sham, WT-TAC, *Nod1*$^{-/-}$-Sham and *Nod1*$^{-/-}$-TAC mice. (C) Relative quantification of extracellular matrix proteins (Col18a1, Col14a1, Fn1 and Postn) in LA tissue from WT-Sham (n = 4), WT-TAC (n = 4), *Nod1*$^{-/-}$-Sham (n = 4) and *Nod1*$^{-/-}$-TAC (n = 5) mice. Zq values represent log- fold change in protein abundance relative to WT-Sham, standardized to units of standard deviation. Differences were considered statistically significant at FDR < 0.05. (D) LA mRNA expression of *Tgfb1*, normalized to 36B4 and expressed relative to WT-Sham.

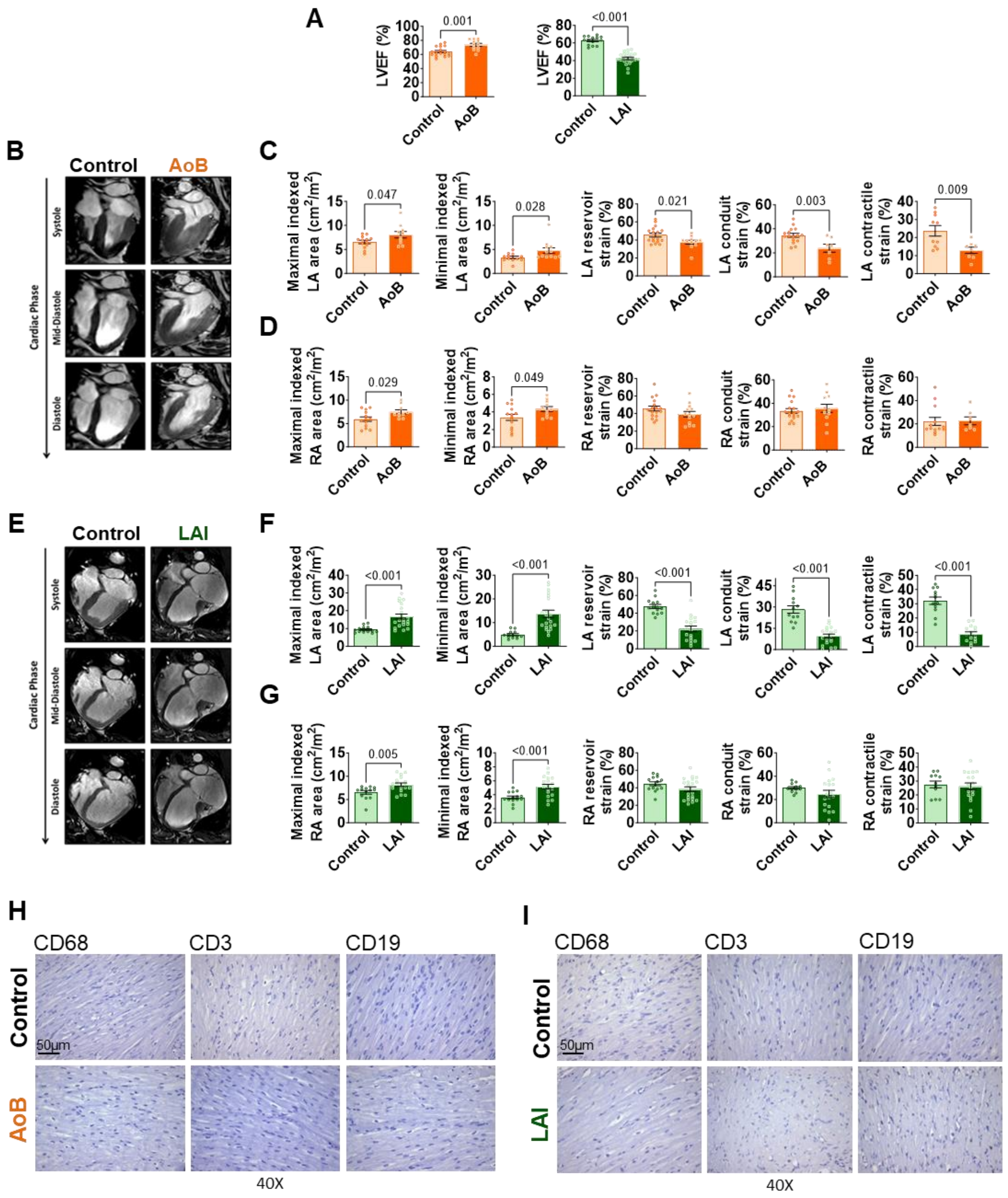

Figure S4. Structural, functional, and immunohistological characterization of atrial remodeling in AoB and LAI porcine models. (A) LVEF in Control (*n* = 17) and AoB (*n* = 12) Yucatan minipigs, and in Control (*n* = 14) and LAI (*n* = 21) Large White pigs. (B) Representative CMR images from Control and AoB pigs. (C-D) Maximal and minimal LA (C) and RA (D) areas and reservoir, conduit, and contractile strain in Control (*n* = 14-17) and AoB

(n = 21) groups. (E) Representative CMR images from Control and LAI pigs. (F-G) Maximal and minimal LA (F) and RA (G) areas and reservoir, conduit, and contractile strain in Control (n = 10-17) and LAI (n = 7-12) groups. (H-I) IHC staining for CD68, CD3, and CD19 in LA tissue from (H) AoB and (I) LAI porcine models. Data are mean ± SEM; statistical significance determined by unpaired t-test ($P < 0.05$).

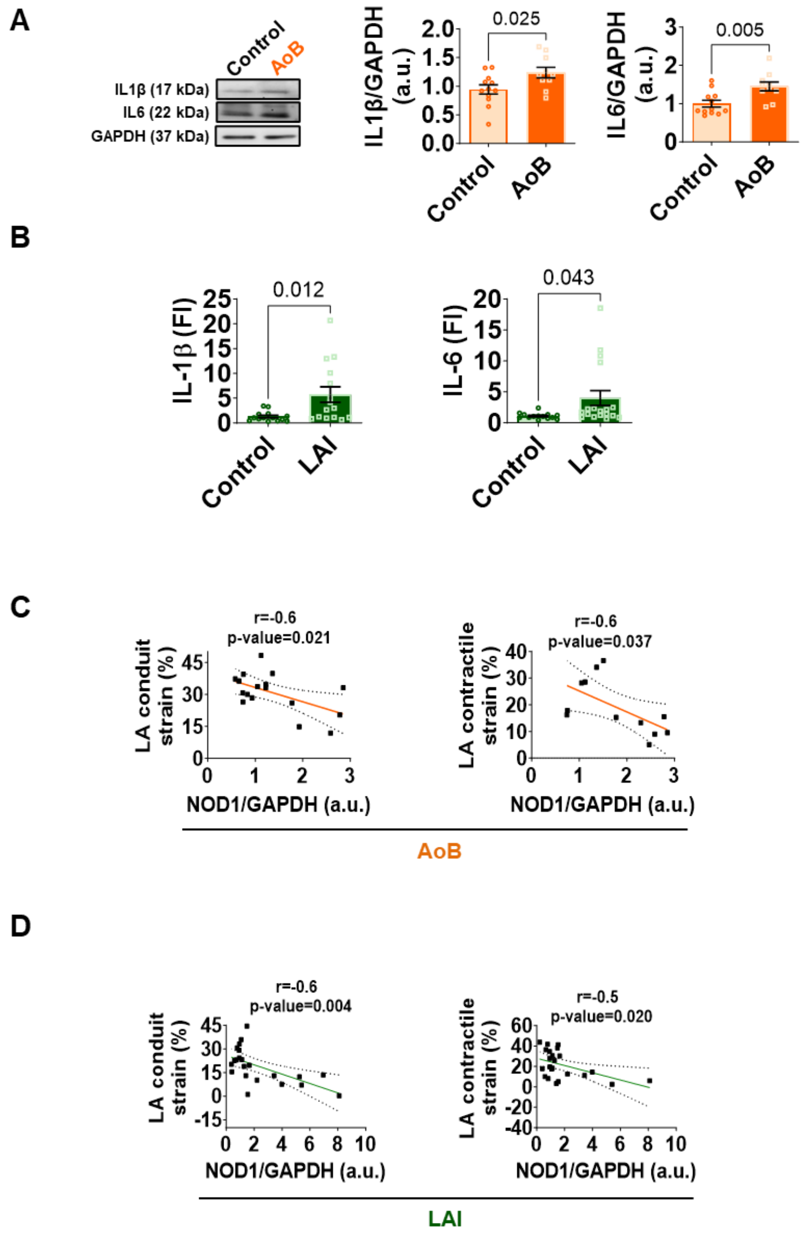

Figure S5. Inflammatory cytokine expression and NOD1–strain associations in AoB and LAI porcine atrial remodeling. (A) Western blot and quantification of IL-1β and IL-6 in LA tissue from Control (n = 12) and AoB (n = 10) Yucatan minipigs, normalized to GAPDH. (B) mRNA levels of IL1β and IL6 in LA tissue from Control and LAI Large White pigs, normalized to GAPDH and relative to the Control group (Control, n = 13; LAI, n = 17). (C-D) Correlations between LA NOD1 protein levels and conduit and contractile strain in AoB (C) and LAI (D) porcine models. Data are mean ± SEM; statistical analyses performed by unpaired t-test or Pearson's correlation ($P < 0.05$ considered significant).

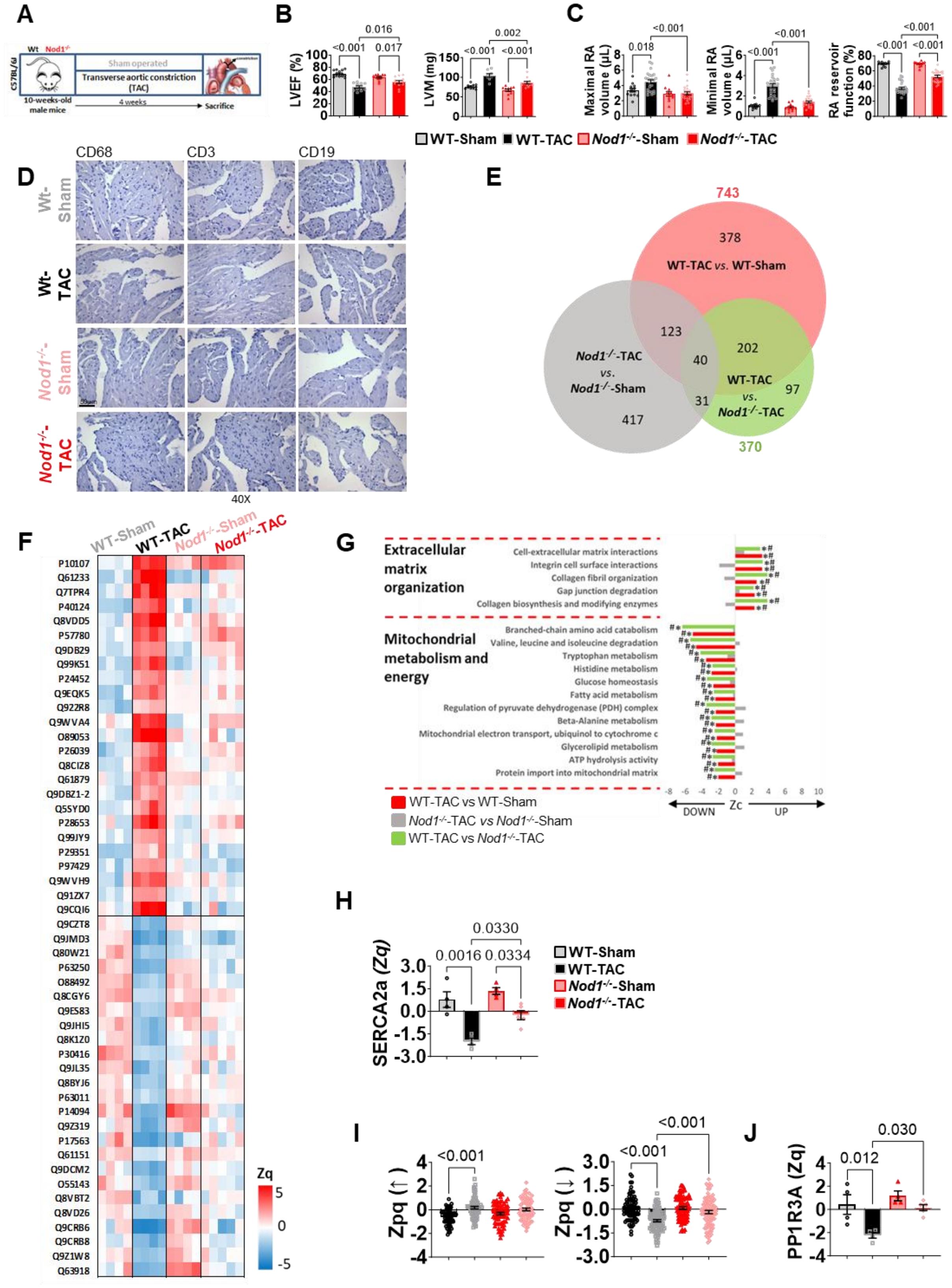

Figure S6. Structural and functional characterization and proteomic profiling in WT and *Nod1*-/- mice subjected to TAC. (A) Schematic of TAC in WT and *Nod1*-/- mice. (B) LVEF

and left ventricular mass (LVM) in WT-Sham (n = 15), WT-TAC (n = 12), *Nod1*$^{-/-}$-Sham (n = 11) and *Nod1*$^{-/-}$-TAC (n = 12) mice. (C) Maximal and minimal RA volumes and reservoir function in all groups. Data are mean ± SEM; statistical significance determined by ANOVA with Tukey's post hoc ($P < 0.05$). (D) IHC staining of LA tissue from WT and *Nod1*$^{-/-}$ Sham and TAC mice for CD68, CD3, and CD19. (E) Venn diagram illustrating the overlap of differentially expressed proteins in LA tissue from WT-Sham (n = 4), WT-TAC (n = 4), *Nod1*$^{-/-}$-Sham (n = 4) and *Nod1*$^{-/-}$-TAC (n = 5) mice. (F) Heatmap displaying the top 25 up and downregulated proteins across WT-TAC and the other experimental groups. Zq values represent $\log_2$ fold-change in protein abundance relative to WT-Sham, standardized to units of standard deviation. Proteins were selected based on a significance threshold of FDR < 0.05, and only those quantified with more than one peptide were included. (G) Functional enrichment analysis of murine LA tissue of extracellular matrix organization and mitochondrial metabolism and energy-related pathways in all groups. Zc represents $\log_2$ fold-change standardized to SD. Significant differences noted at FDR<0.05 (*WT-TAC vs WT-Sham; #WT-TAC vs *Nod1*$^{-/-}$-TAC). (H) Relative quantification of SERCA2a. (I) Phosphopeptide abundance (Zpq) for higher and lower phosphopeptides. Zpq values are reported as $\log_2$ fold-change relative to WT-Sham replicates, expressed in units of standard deviation. (J) Relative quantification of PP1R3A in LA tissue from WT-Sham (n = 4), WT-TAC (n = 4), *Nod1*$^{-/-}$-Sham (n = 4) and *Nod1*$^{-/-}$-TAC (n = 5) mice. Zq values represent log2 fold-change in protein abundance relative to WT-Sham, standardized to units of standard deviation. Differences were considered statistically significant at FDR<0.05.

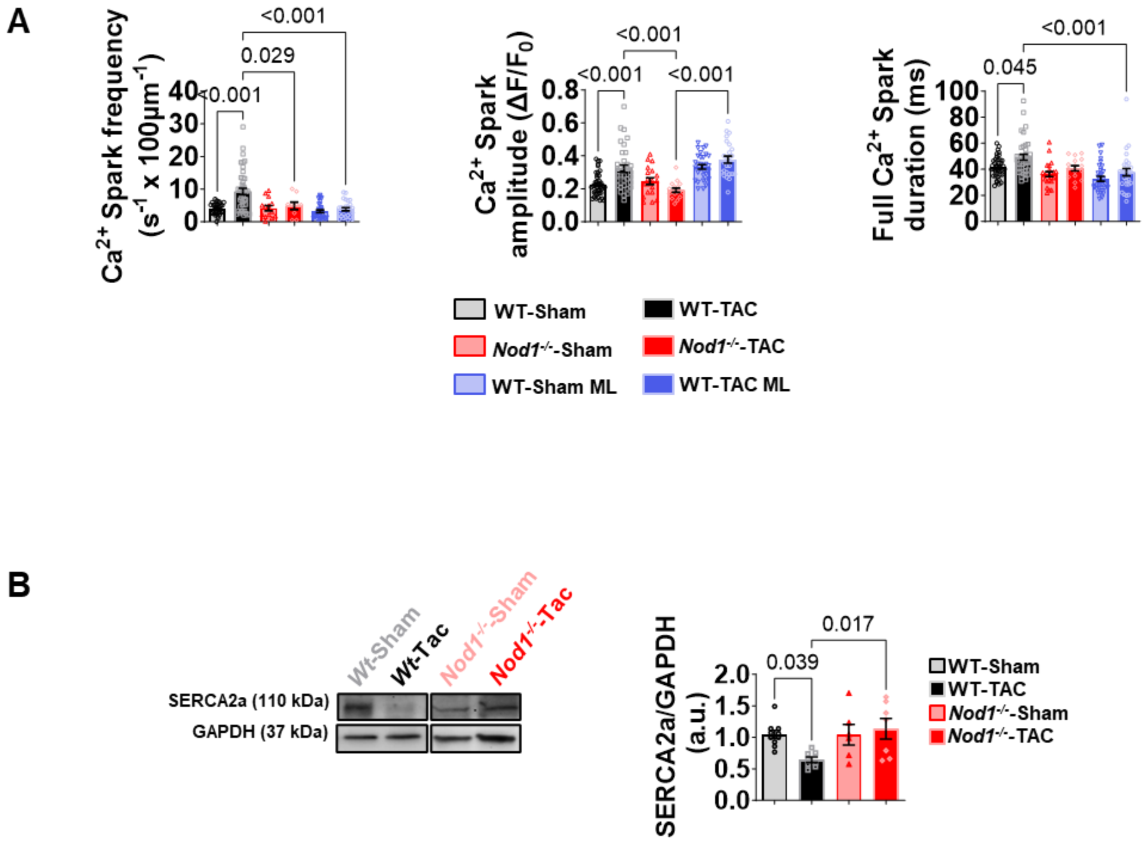

Figure S7. NOD1 mediates TAC-induced alterations in Ca^{2+} spark properties and SERCA2a downregulation in WT mice. (A) Ca^{2+} spark frequency, amplitude and full duration in LA cardiomyocytes from WT-Sham (n = 33-41 cells/6 mice), WT-TAC (n = 19-29 cells/5 mice), *Nod1*$^{-/-}$-Sham (n = 14-23 cells/4 mice), *Nod1*$^{-/-}$-TAC (n = 8-12 cells/5 mice), WT-Sham ML (n = 32-33 cells/3 mice), and WT-TAC ML (n = 26-29 cells/3 mice). (B) Western blot and quantification of SERCA2a in LA tissue from WT-Sham (n = 10), WT-TAC (n = 8), *Nod1*$^{-/-}$-Sham (n = 6) and *Nod1*$^{-/-}$-TAC (n = 7) mice, normalized to GAPDH. Data are mean ± SEM; statistical significance determined by ANOVA with Tukey's post hoc ($P < 0.05$).

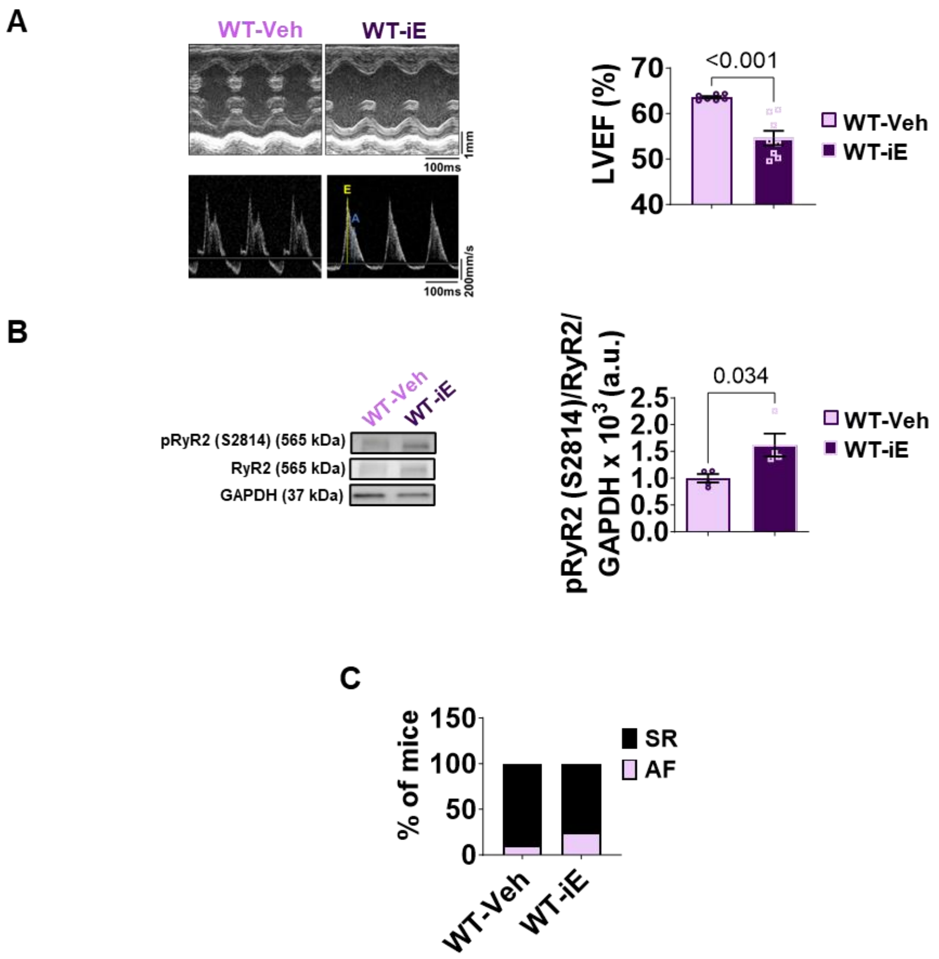

Figure S8. NOD1 activation induces cardiac dysfunction and RyR2 phosphorylation in WT mice. WT mice were treated with the NOD1 agonist C12-iE-DAP (iE; 3.3 mg/kg i.p. 3 days). (A) Representative echocardiographic images (left panel) and LVEF (right panel) in WT-Vehicle (WT-Veh, *n* = 7) and WT-iE (*n* = 8) mice. (B) Western blot and quantification of RyR2 phosphorylation at Ser2814 (CaMKII site) in atrial tissue, normalized to total RyR2 and GAPDH (WT-Veh, *n* = 4; WT-iE, *n* = 4). (C) Atrial fibrillation (AF) inducibility expressed as percentage of animals with induced AF per group in an additional cohort of WT-Veh (n = 7) and WT-iE (n = 7) mice. Data are mean ± SEM; statistical significance determined by unpaired t-test or chi-square test ($P < 0.05$ considered significant).

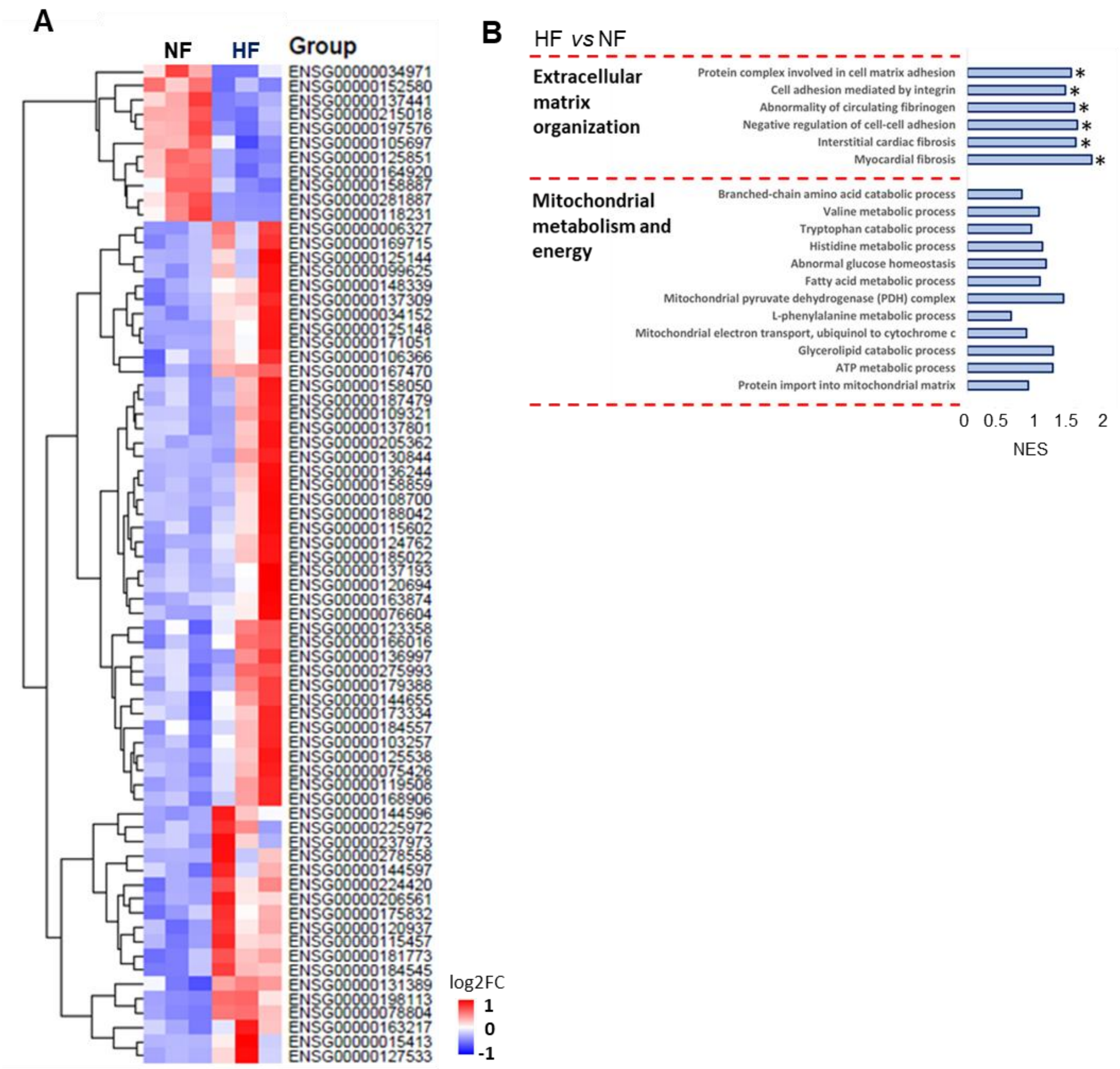

Figure S9. Transcriptomic and molecular alterations in atrial myocardium from NF and HF patients. (A) Hierarchical heatmap of differentially expressed genes in atrial tissue from HF *vs* NF patients. (B) Transcriptomic gene set enrichment analysis of extracellular matrix organization and mitochondrial metabolism and energy-related pathways in both groups. * denoted significantly enriched pathways at FDR < 0.05.
